# Supplementary figures and images for: Domestication reshaped the genetic basis of inbreeding depression in a maize landrace compared to its wild relative, teosinte
Source: PLoS Genet. 2021 Dec 20;17(12):e1009797. doi: 10.1371/journal.pgen.1009797 (PMC8722731; doi:10.1371/journal.pgen.1009797)

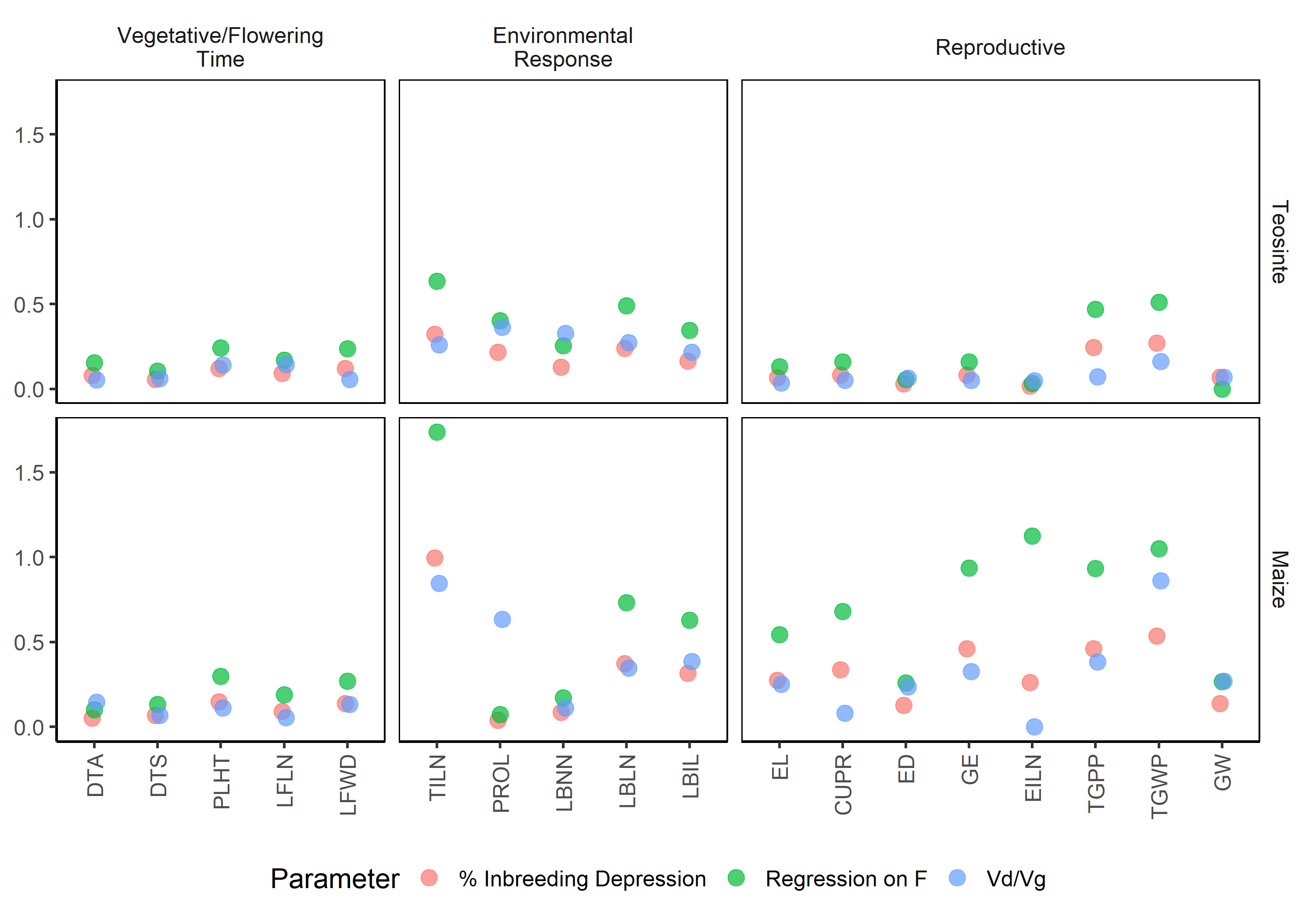

Supplement: S1 Fig — (PNG) [file pgen.1009797.s001.png]

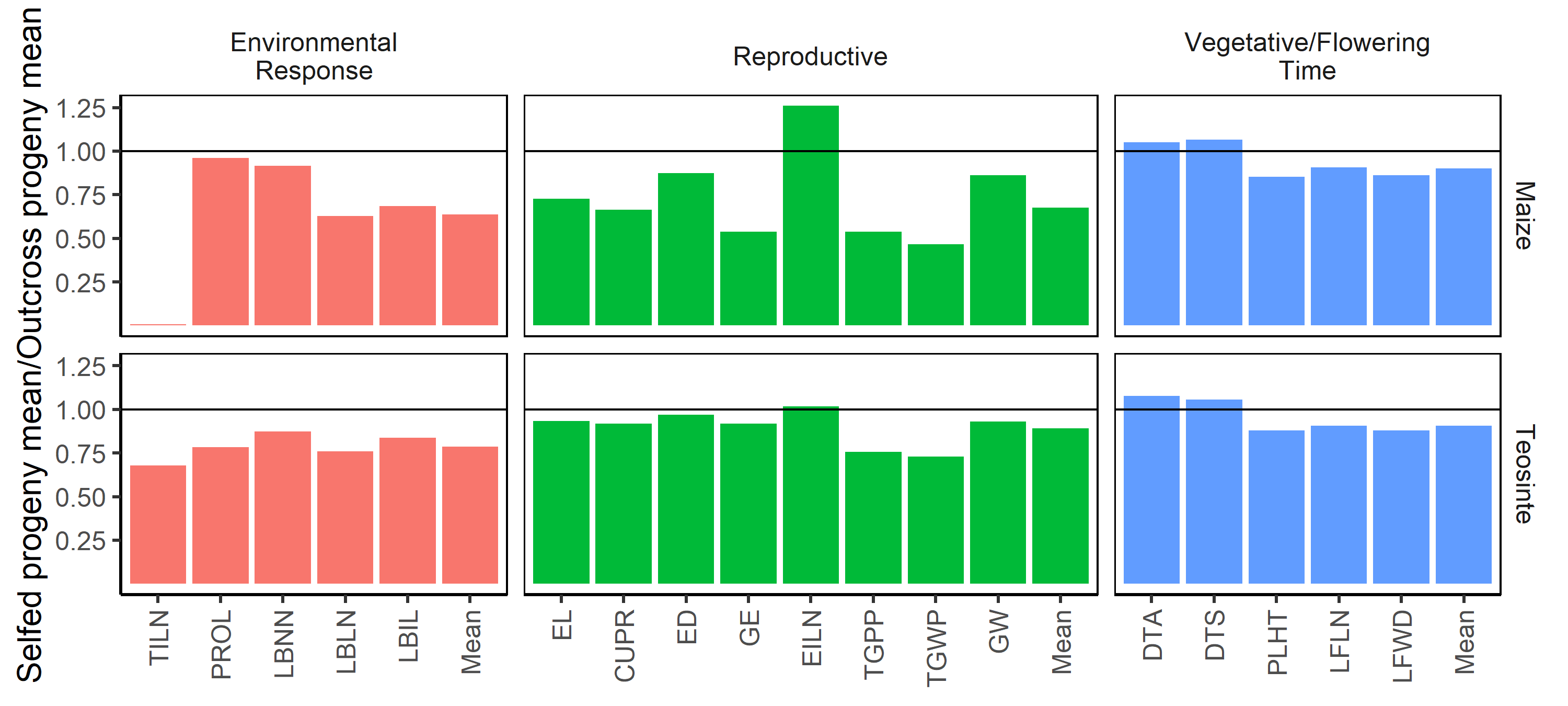

Supplement: S2 Fig — (PNG) [file pgen.1009797.s002.png]

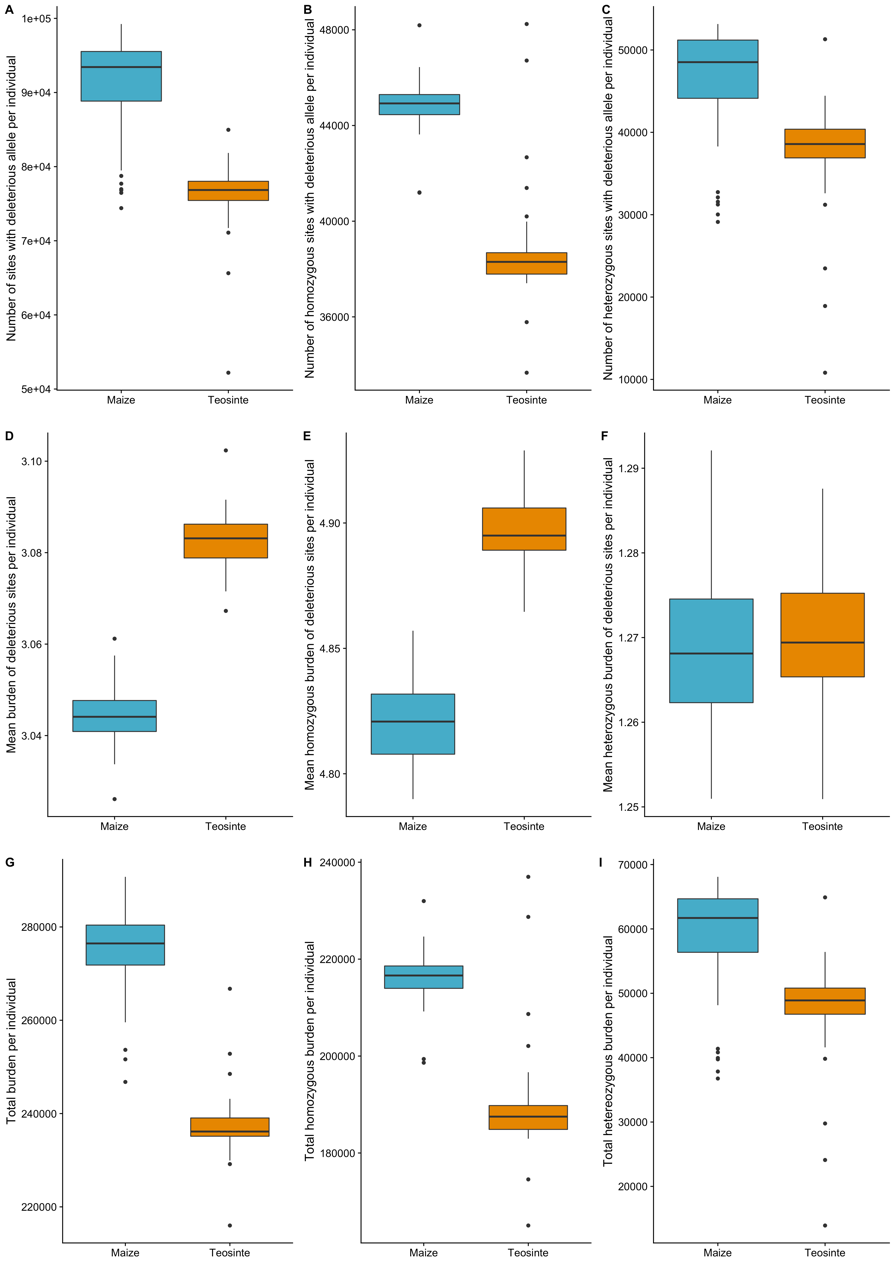

Supplement: S3 Fig — (A) Total segregating deleterious sites per individual parent, (B) homozygous deleterious sites per parent, (C) Heterozygous deleterious sites per parent, (D) Mean burden per site per parent based on genomic evolutionary rate profiling (GERP) score under a model of partial recessivity, including only sites segregating within the parent’s population, (E) Mean homozygous burden per parent, (F) Mean heterozygous burden per parent, (G) Total burden per parent based on GERP scores, (H) Total homozygous burden per parent based on GERP scores, (I) Total heterozygous burden per parent. (PNG) [file pgen.1009797.s003.png]

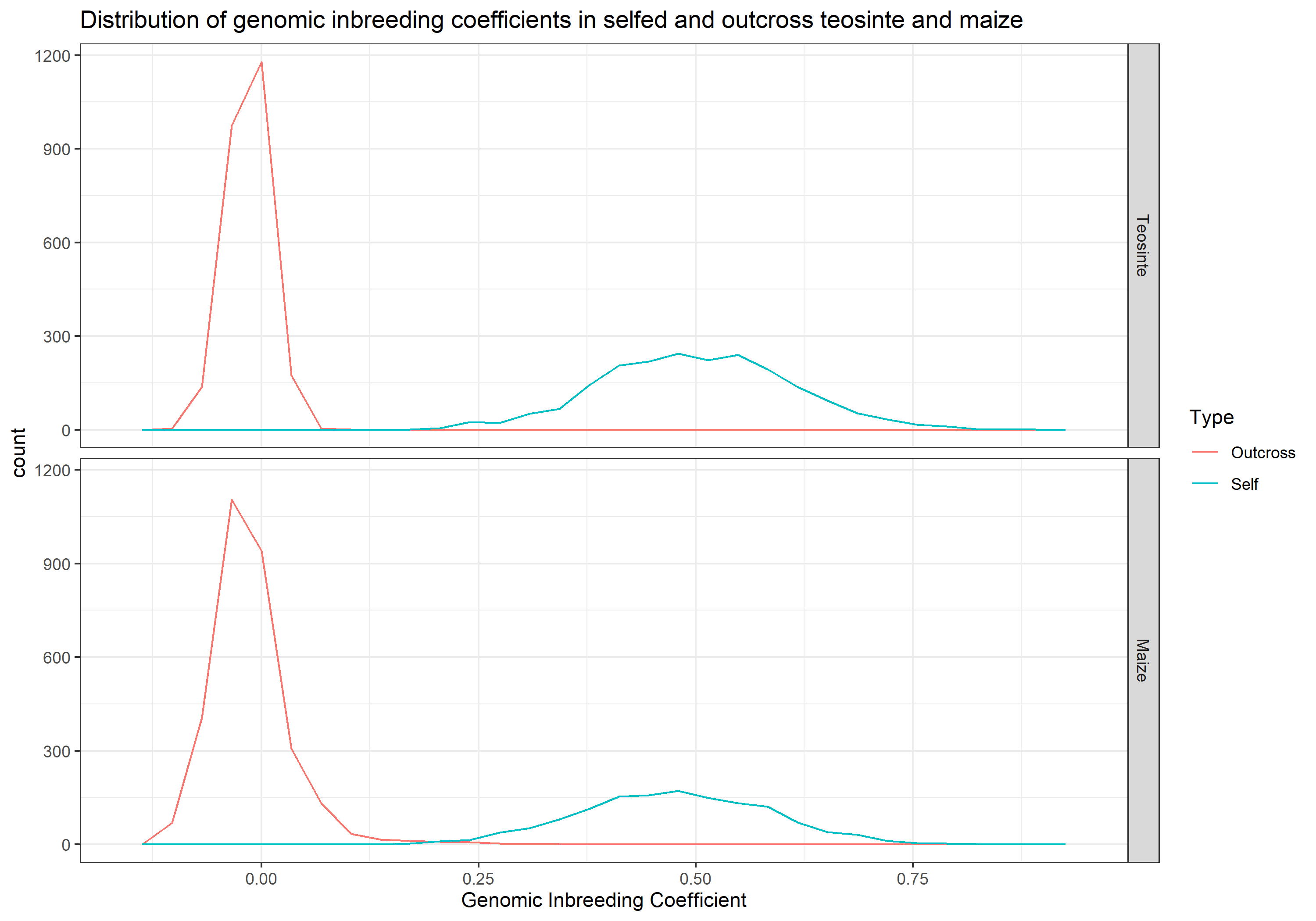

Supplement: S4 Fig — (PNG) [file pgen.1009797.s004.png]

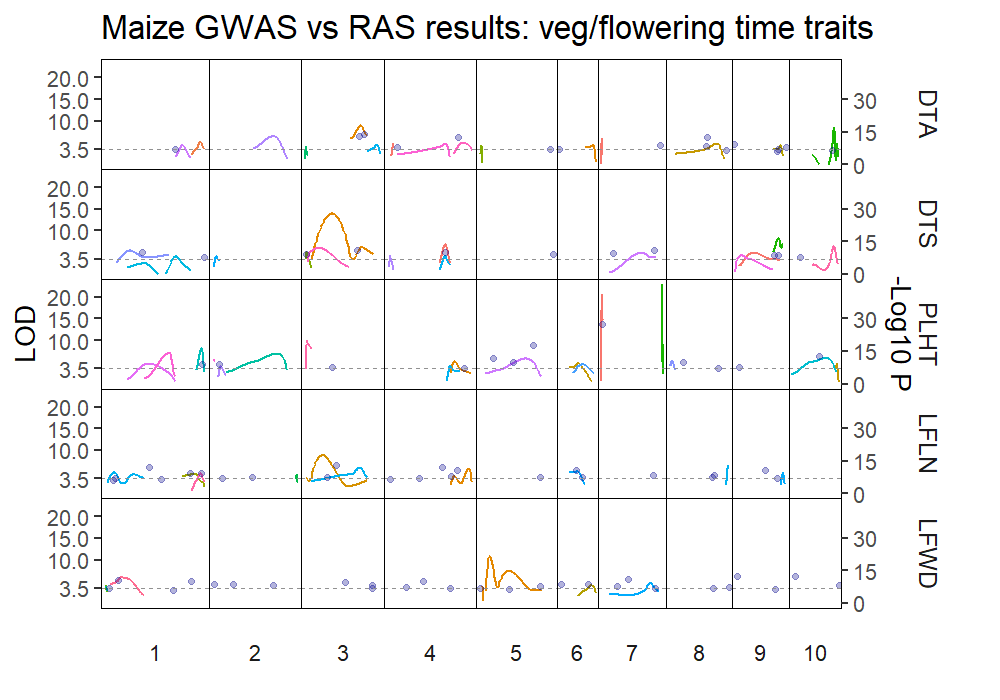

Supplement: S5 Fig — Each row of figures corresponds to one trait. Each column corresponds to one of the ten chromosome pairs in maize. Logarithm of odds (LOD) scores for QTL models are plotted for the 2-LOD support interval for each QTL. LOD curves correspond to the effects of a single parental haplotype, and different parent effects are plotted with different colors. Blue dots represent the -log10 p-values of significant GWAS associations from Chen et al. [44]. (PNG) [file pgen.1009797.s005.png]

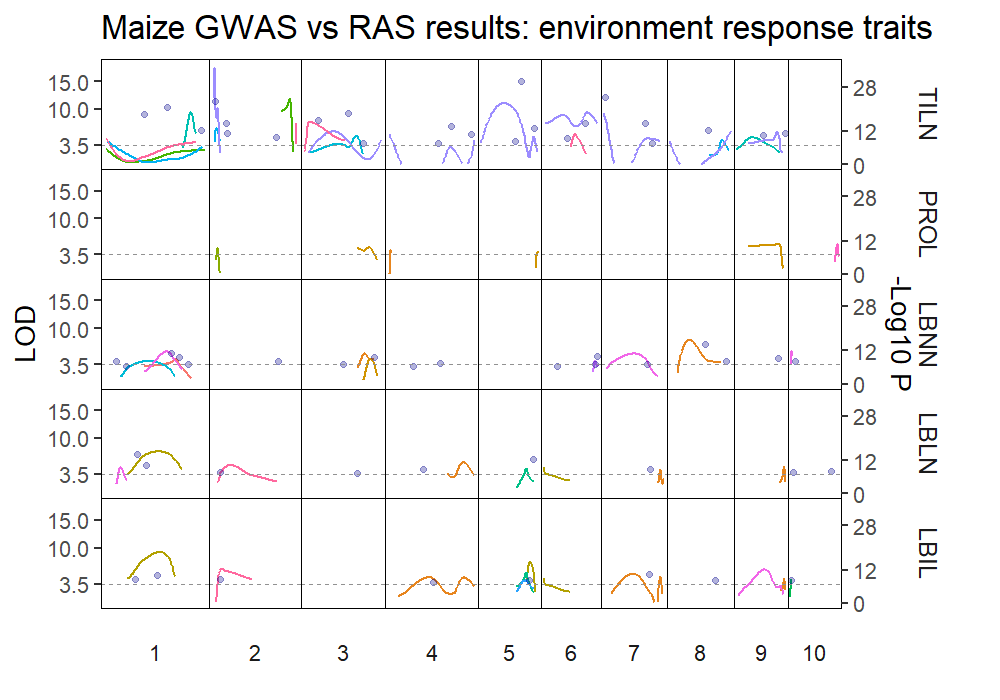

Supplement: S6 Fig — Each row of figures corresponds to one trait. Each column corresponds to one of the ten chromosome pairs in maize. Logarithm of odds (LOD) scores for QTL models are plotted for the 2-LOD support interval for each QTL. LOD curves correspond to the effects of a single parental haplotype, and different parent effects are plotted with different colors. Blue dots represent the -log10 p-values of significant GWAS associations from Chen et al. [44]. (PNG) [file pgen.1009797.s006.png]

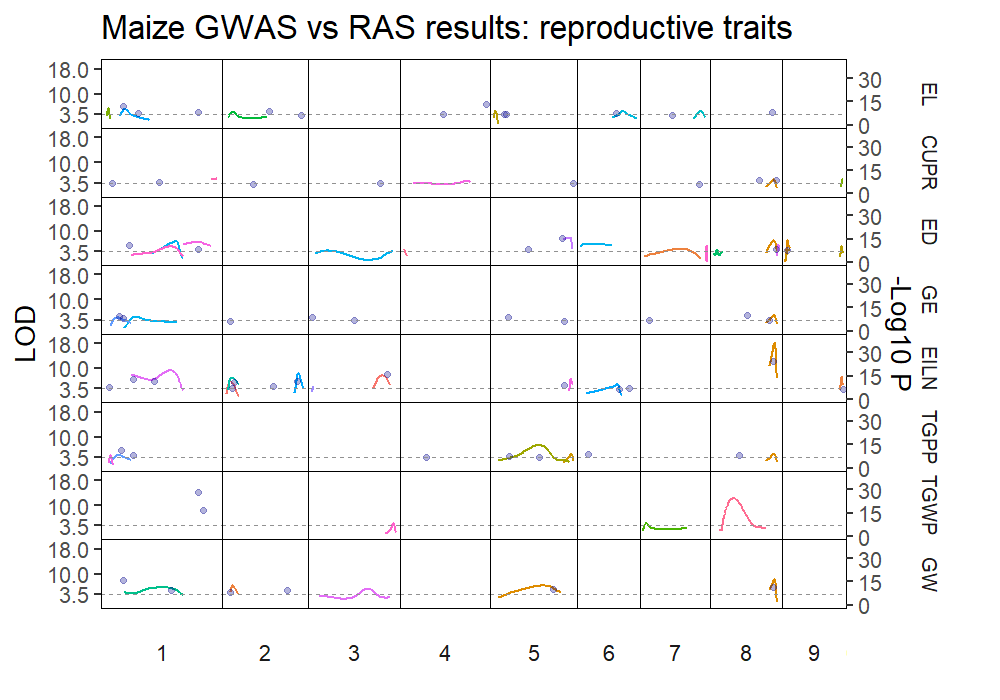

Supplement: S7 Fig — Each row of figures corresponds to one trait. Each column corresponds to one of the ten chromosome pairs in maize. Logarithm of odds (LOD) scores for QTL models are plotted for the 2-LOD support interval for each QTL. LOD curves correspond to the effects of a single parental haplotype, and different parent effects are plotted with different colors. Blue dots represent the -log10 p-values of significant GWAS associations from Chen et al. [44]. (PNG) [file pgen.1009797.s007.png]

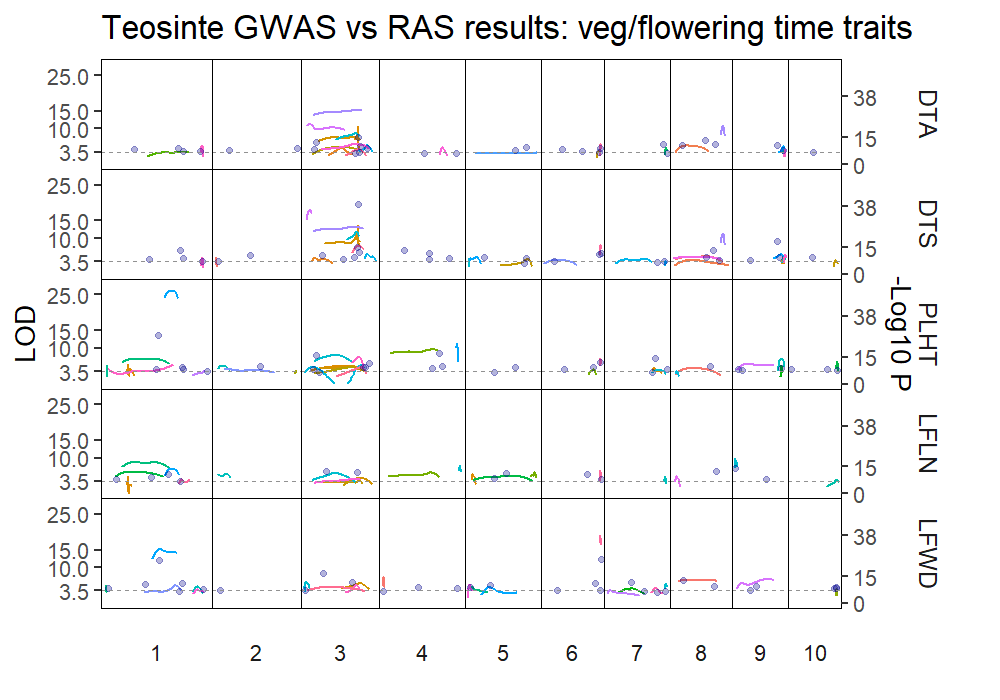

Supplement: S8 Fig — Each row of figures corresponds to one trait. Each column corresponds to one of the ten chromosome pairs in maize/teosinte. Logarithm of odds (LOD) scores for QTL models are plotted for the 2-LOD support interval for each QTL. LOD curves correspond to the effects of a single parental haplotype, and different parent effects are plotted with different colors. Blue dots represent the -log10 p-values of significant GWAS associations from Chen et al. [44]. (PNG) [file pgen.1009797.s008.png]

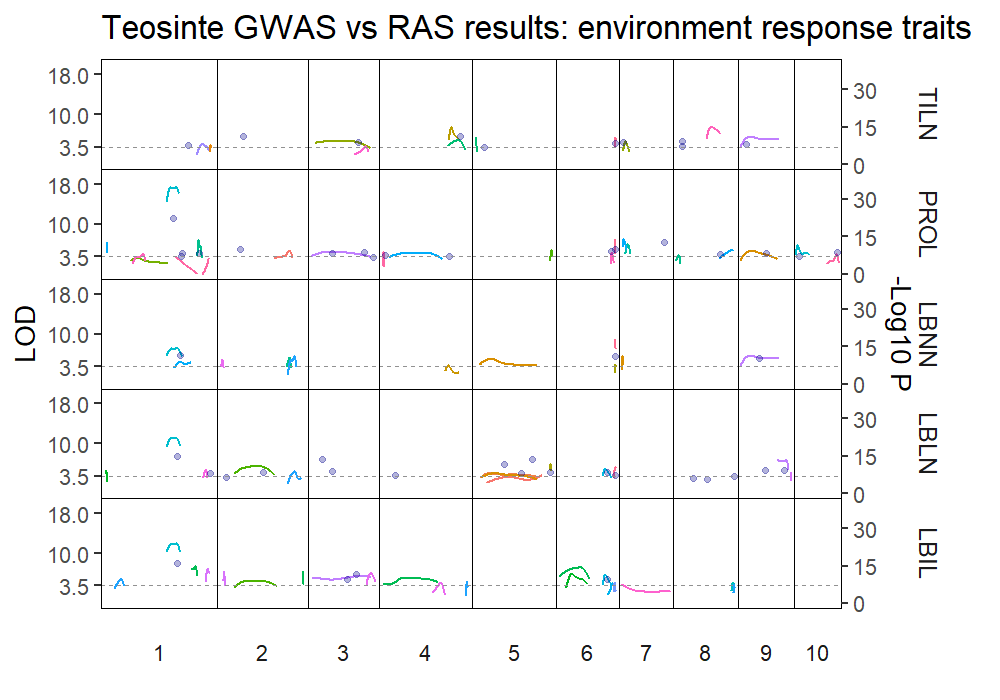

Supplement: S9 Fig — Each row of figures corresponds to one trait. Each column corresponds to one of the ten chromosome pairs in maize/teosinte. Logarithm of odds (LOD) scores for QTL models are plotted for the 2-LOD support interval for each QTL. LOD curves correspond to the effects of a single parental haplotype, and different parent effects are plotted with different colors. Blue dots represent the -log10 p-values of significant GWAS associations from Chen et al. [44]. (PNG) [file pgen.1009797.s009.png]

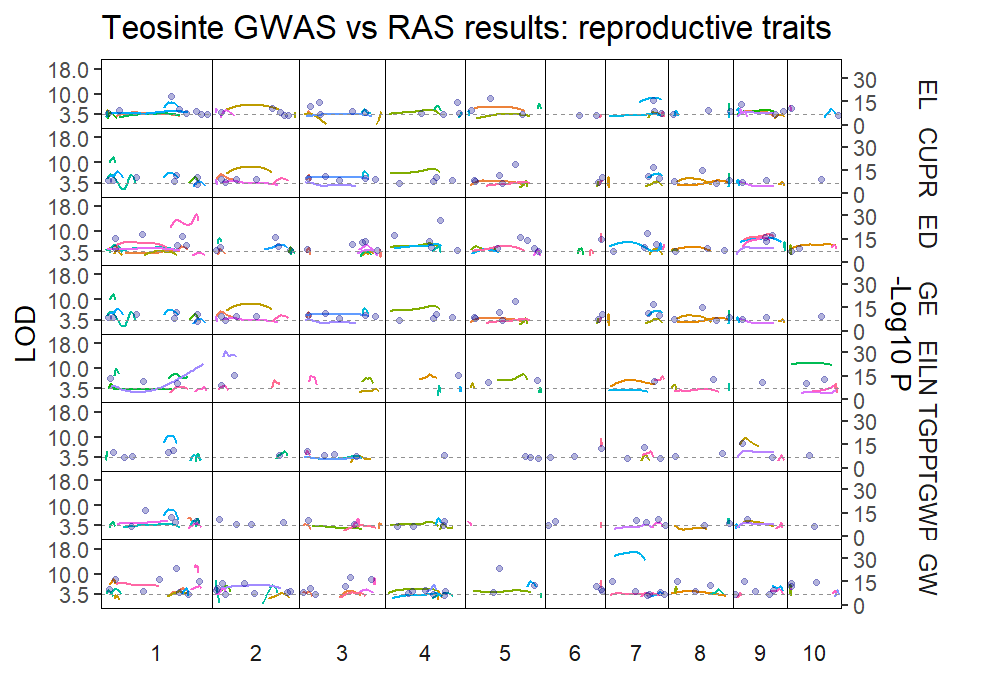

Supplement: S10 Fig — Each row of figures corresponds to one trait. Each column corresponds to one of the ten chromosome pairs in maize/teosinte. Logarithm of odds (LOD) scores for QTL models are plotted for the 2-LOD support interval for each QTL. LOD curves correspond to the effects of a single parental haplotype, and different parent effects are plotted with different colors. Blue dots represent the -log10 p-values of significant GWAS associations from Chen et al. [44]. (PNG) [file pgen.1009797.s010.png]

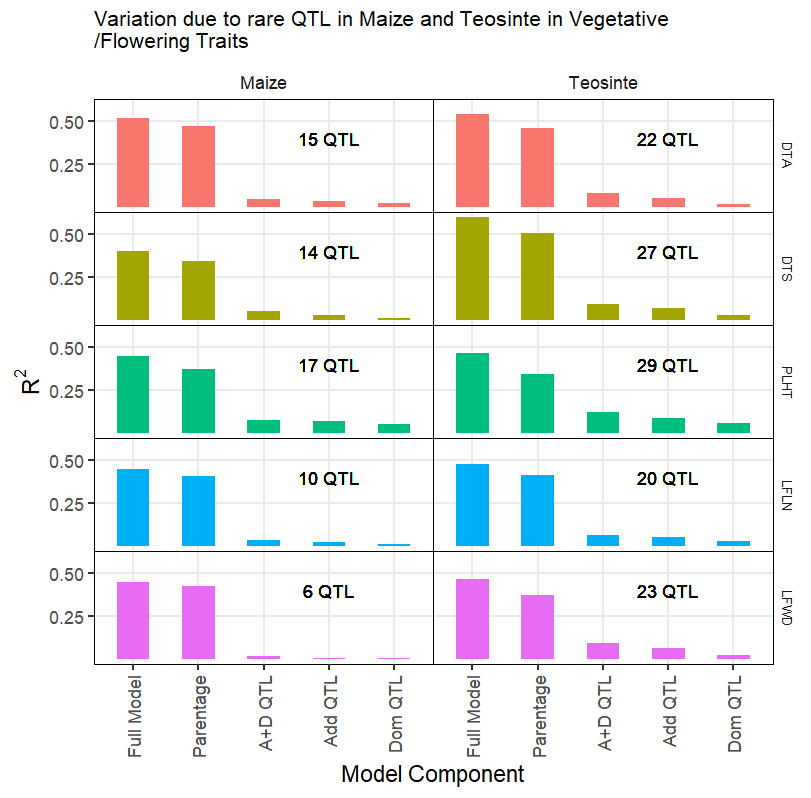

Supplement: S11 Fig — Full model includes parentage, QTL additive, and QTL dominance effects. The proportion of variance due specifically to parentage, additive plus dominance QTL effects, additive QTL effects only, or dominance QTL effects only was estimated by measuring the decrease in R2 after removing one of those factors from the full model. (PNG) [file pgen.1009797.s011.png]

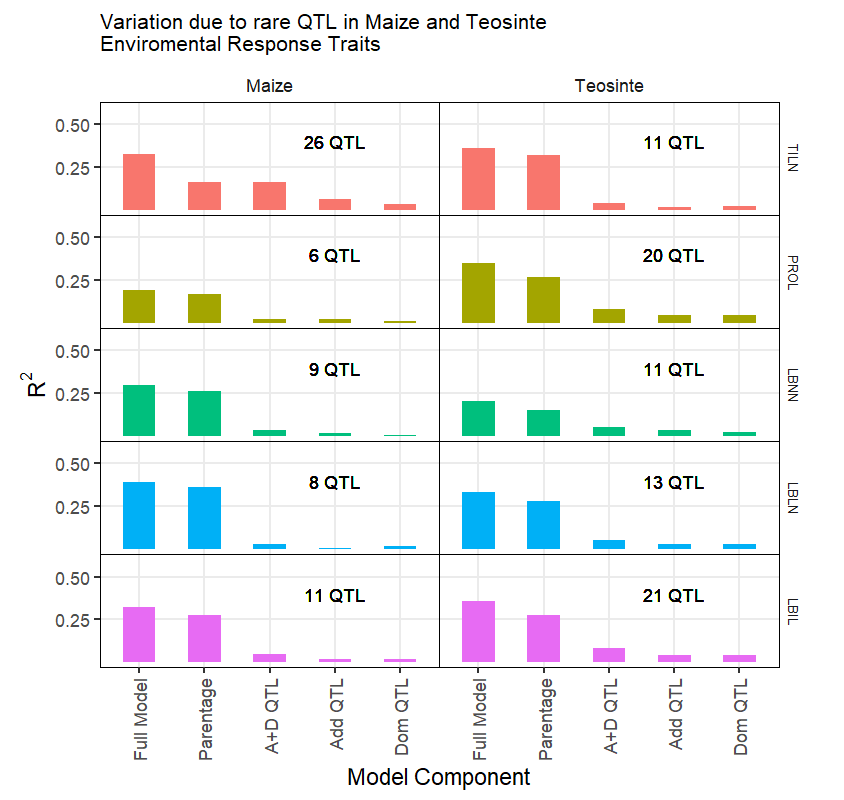

Supplement: S12 Fig — Full model includes parentage, QTL additive, and QTL dominance effects. The proportion of variance due specifically to parentage, additive plus dominance QTL effects, additive QTL effects only, or dominance QTL effects only was estimated by measuring the decrease in R2 after removing one of those factors from the full model. (PNG) [file pgen.1009797.s012.png]

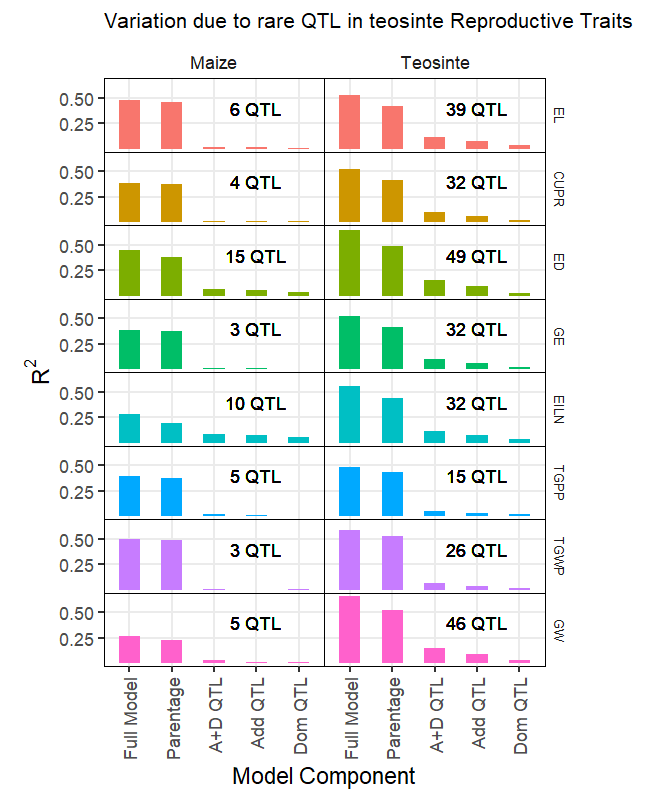

Supplement: S13 Fig — Full model includes parentage, QTL additive, and QTL dominance effects. The proportion of variance due specifically to parentage, additive plus dominance QTL effects, additive QTL effects only, or dominance QTL effects only was estimated by measuring the decrease in R2 after removing one of those factors from the full model. (PNG) [file pgen.1009797.s013.png]

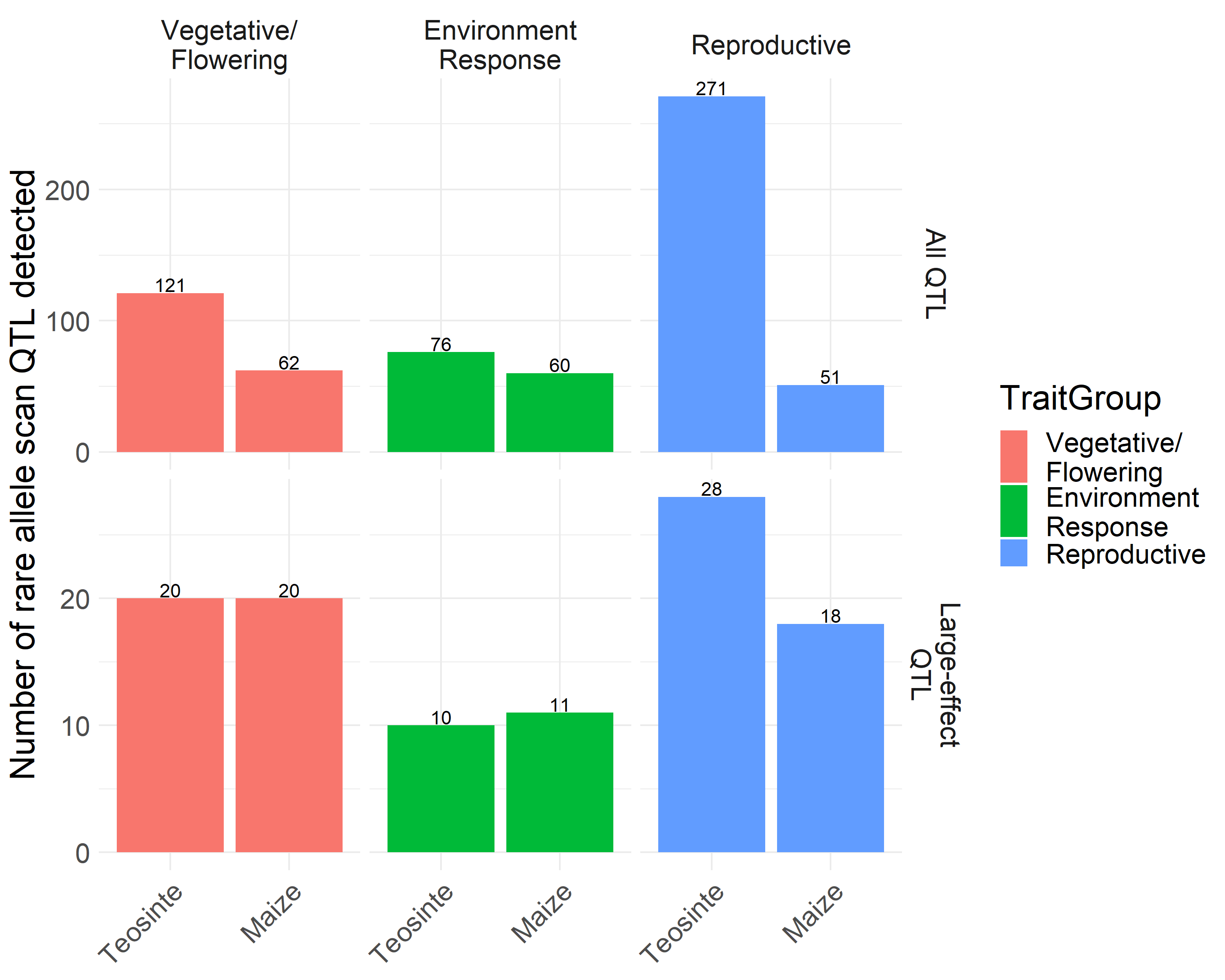

Supplement: S14 Fig — Number of large-effect (> 1 phenotypic standard deviation effect) rare allele scan QTL detected per trait group and population. (PNG) [file pgen.1009797.s014.png]

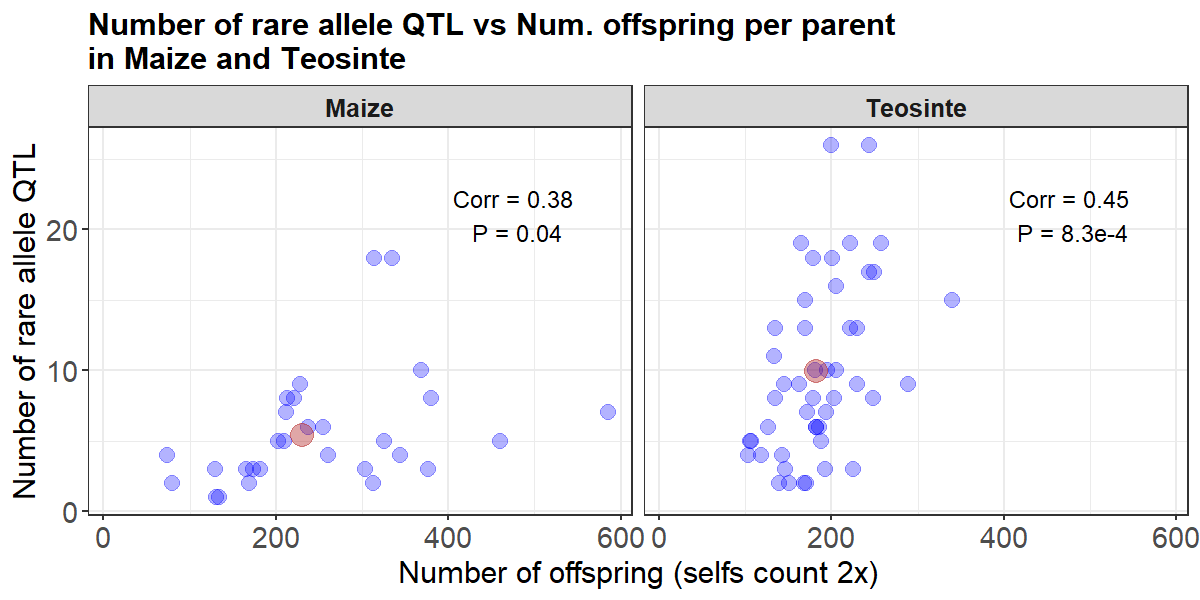

Supplement: S15 Fig — (PNG) [file pgen.1009797.s015.png]

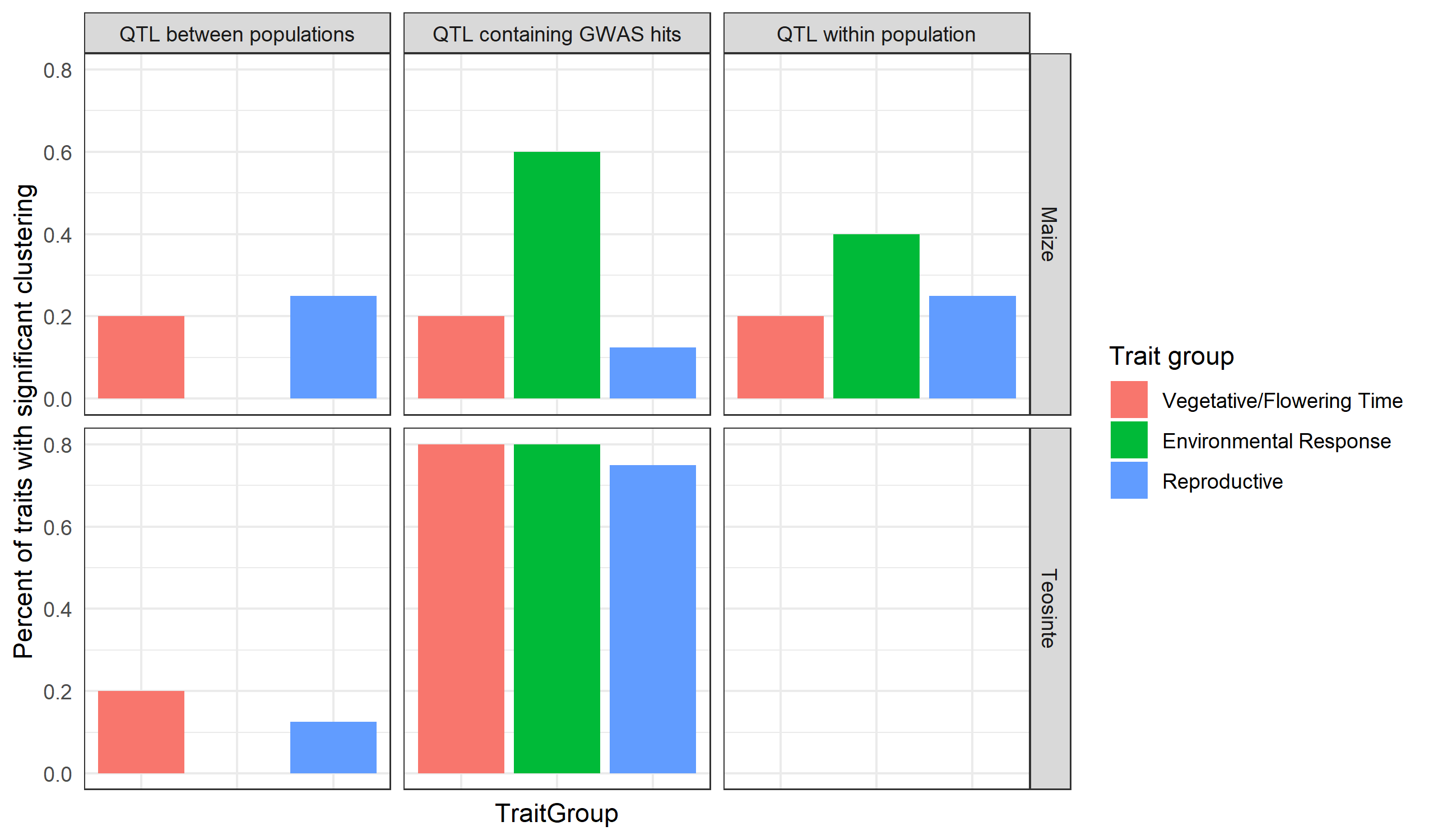

Supplement: S16 Fig — (PNG) [file pgen.1009797.s016.png]

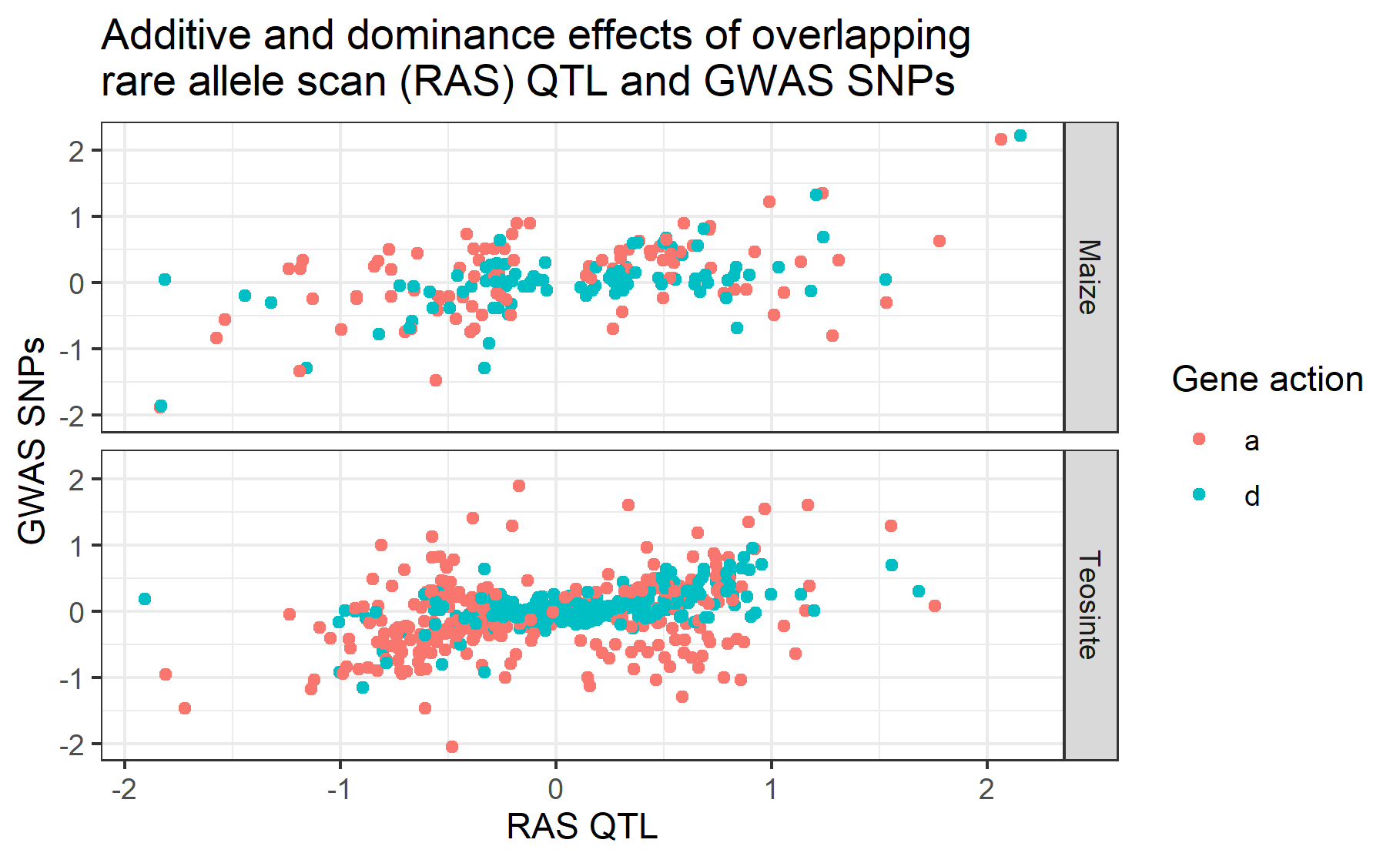

Supplement: S17 Fig — Effects are standardized to the phenotypic standard deviation for each trait and population. (PNG) [file pgen.1009797.s017.png]

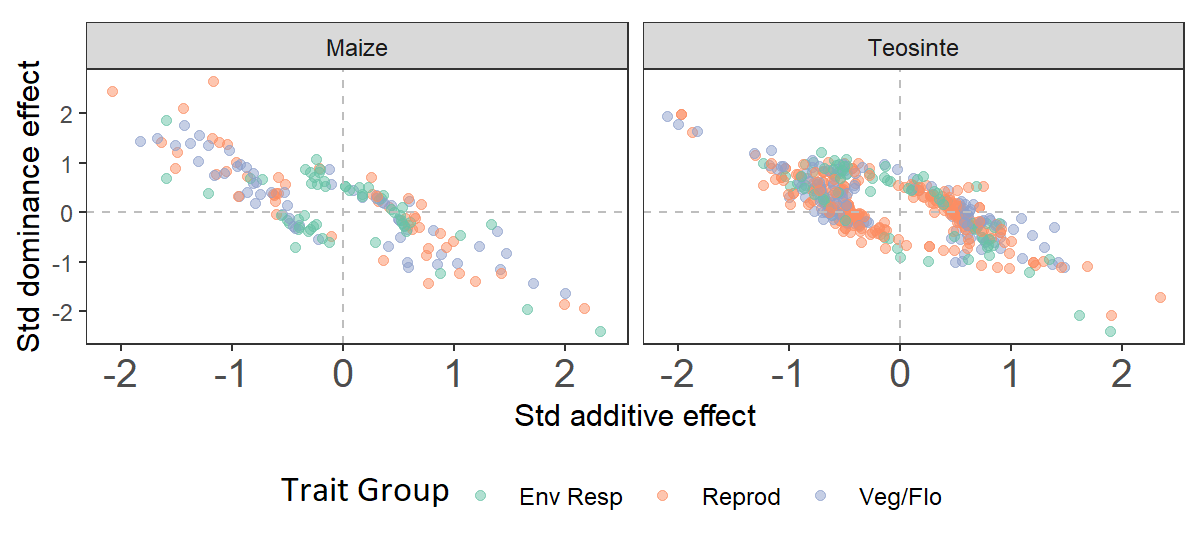

Supplement: S18 Fig — Effects are standardized to the phenotypic standard deviation for each trait and population. (PNG) [file pgen.1009797.s018.png]

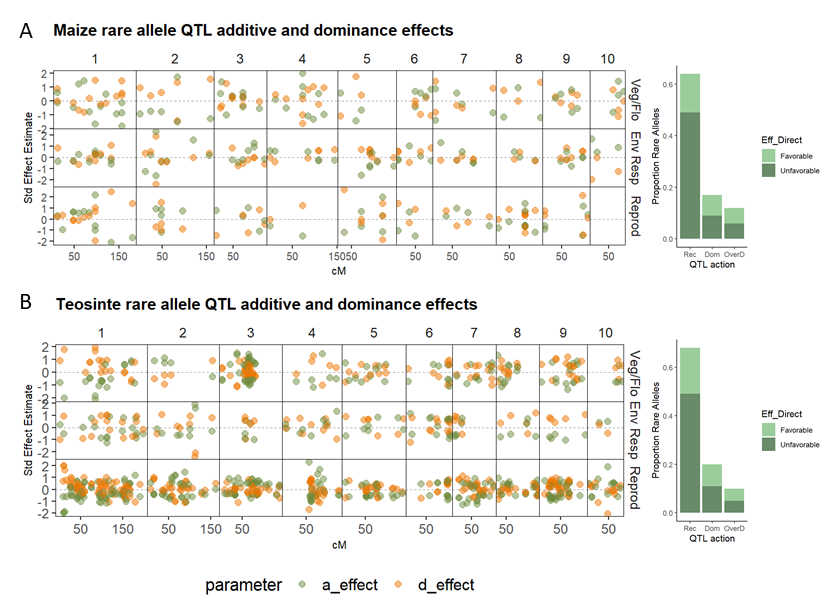

Supplement: S19 Fig — Standardized additive (a) and dominance (d) effects of rare allele scan QTL effects plotted by genome position within each of the ten chromosome pairs for both maize (A) and teosinte (B). Effects are standardized to the phenotypic standard deviation for each trait and population. Effects are plotted within trait categories. Right hand panels (“Eff_Direct”) show the proportion of QTL allele effects where the rare variant effect is favorable (against the direction of inbreeding depression) or unfavorable (in the same direction as inbreeding depression) within categories based on the level of dominance of the rare allele, summed over all traits. Favorable allele effects are (partially to fully) recessive when a > 0 and d < 0, (partially to fully) dominant when a > 0 and d < 0, and overdominant when d > a > 0. Unfavorable alleles are (partially to fully) recessive when a < 0 and d > 0, (partially to fully) dominant when a < 0 and d < 0 alleles, and overdominant when d < a < 0. (PNG) [file pgen.1009797.s019.png]

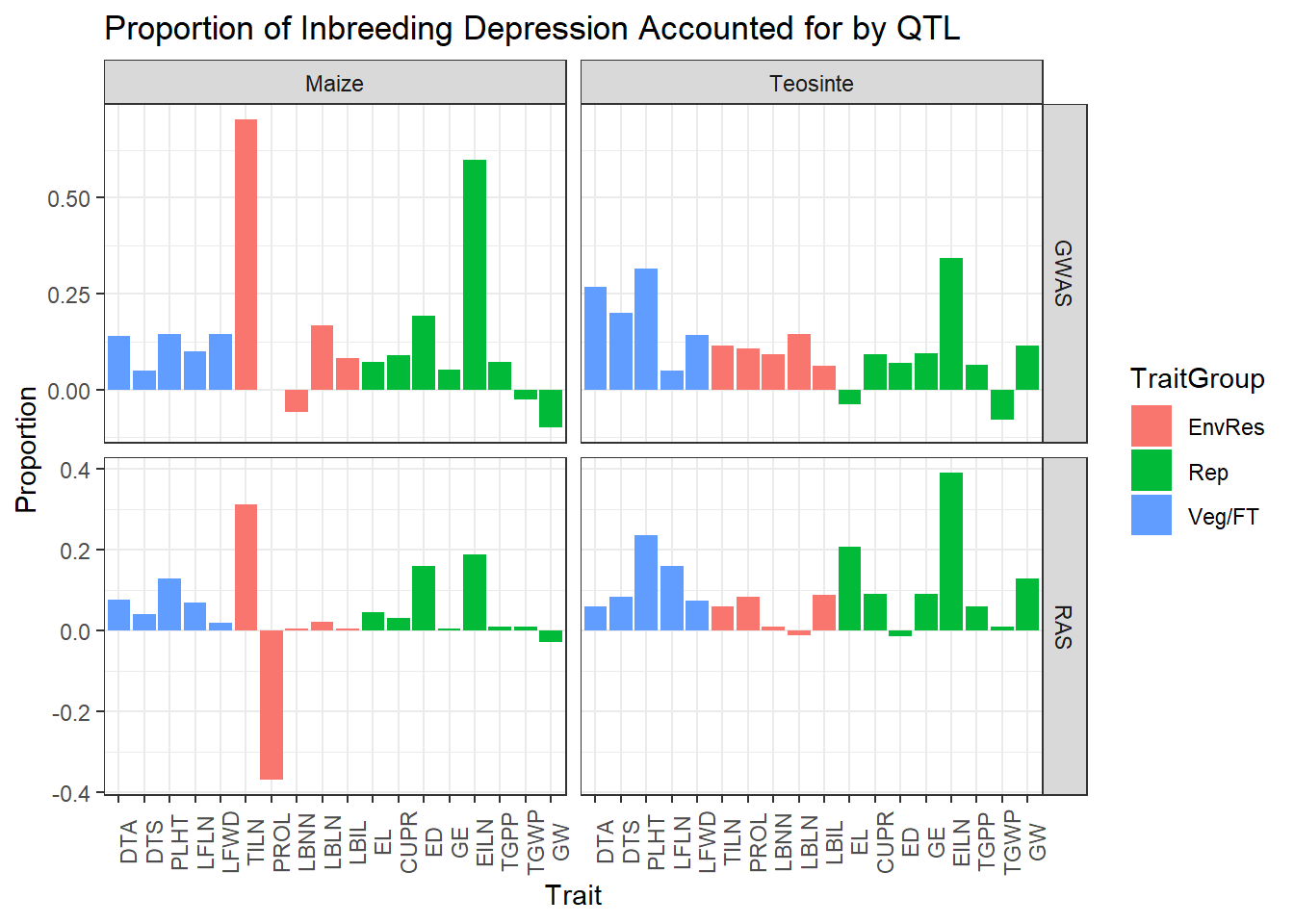

Supplement: S20 Fig — (PNG) [file pgen.1009797.s020.png]

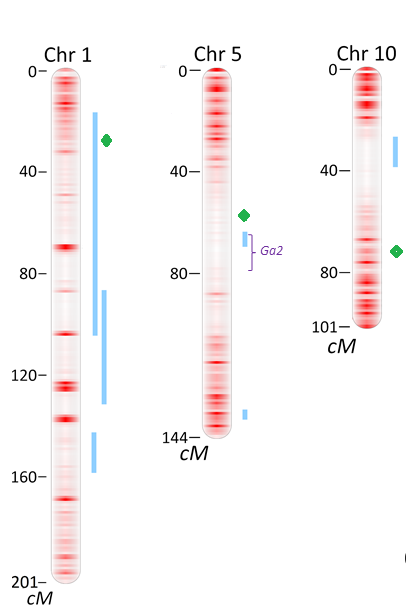

Supplement: S21 Fig — The local recombination rate (cM/Mb) is plotted as intensity of red color for each 1-cM window within either population. The position of gametophyte factor 2 (Ga2) locus is indicated in purple (this locus has not been finely mapped to date). (PNG) [file pgen.1009797.s021.png]

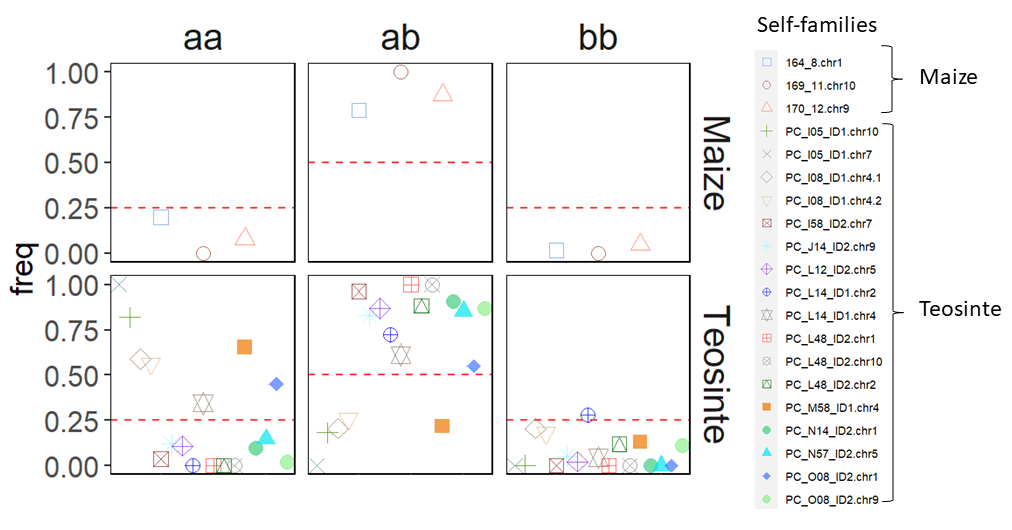

Supplement: S22 Fig — Genotypes aa and bb refer to homozygotes for one of the parental alleles, ab refers to heterozygotes. Shapes refer to particular families. (PNG) [file pgen.1009797.s022.png]

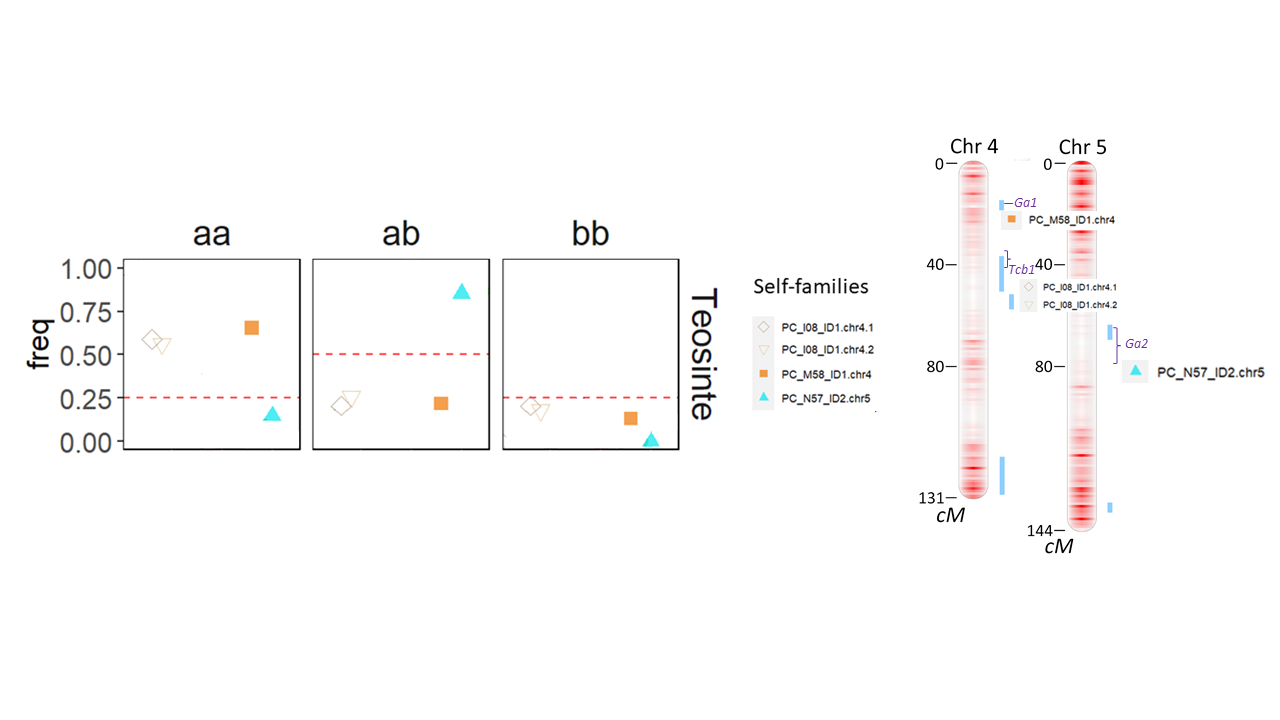

Supplement: S23 Fig — Genotypes aa and bb refer to homozygotes for one of the parental alleles, ab refers to heterozygotes. Shapes refer to particular families. Right hand bar indicates the position of SDR (blue bars) and gametophyte factors (purple) on chromosomes 4 and 5, with the specific parents giving rise to the SDR indicated within their SDR. (PNG) [file pgen.1009797.s023.png]

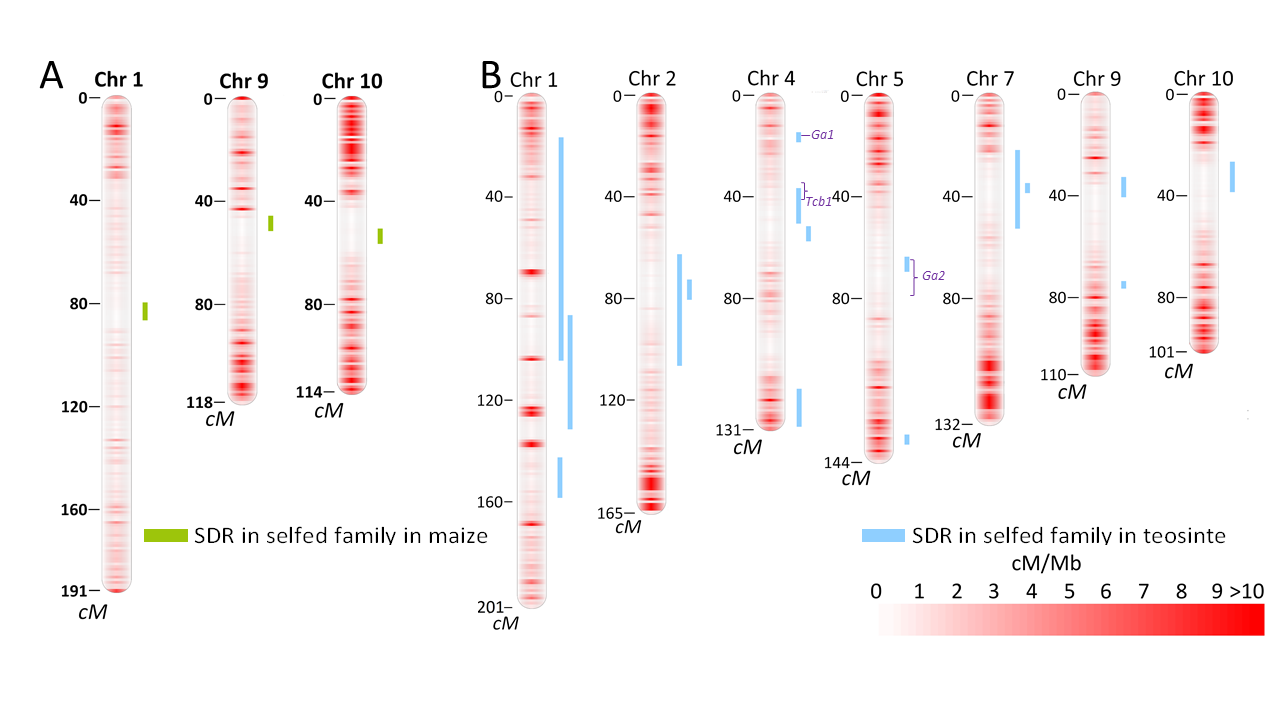

Supplement: S24 Fig — Segregation distortion regions (SDR) superimposed on physical linkage maps of maize (A) or teosinte (B). The local recombination rate (cM/Mb) is plotted as intensity of red color for each 1-cM window within either population. The positions of gametophyte factor 1 (Ga1), gametophyte factor 2 (Ga2), and teosinte crossing barrier 1 (Tcb1) loci are indicated in purple. (PNG) [file pgen.1009797.s024.png]
