# Supplementary material for: Domestication reshaped the genetic basis of inbreeding depression in a maize landrace compared to its wild relative, teosinte
Source: PLoS Genet. 2021 Dec 20;17(12):e1009797. doi: 10.1371/journal.pgen.1009797 (PMC8722731; doi:10.1371/journal.pgen.1009797)
Supplement: Attachment — Submitted filename: pg.corn.9.23.pdf [file pgen.1009797.s041.pdf]

"Domestication Reshaped the Genetic Basis of Inbreeding Depression in a Maize Landrace Compared to its Wild Relative, Teosinte" by Holland et al.

Holland et al present a detailed quantitative genetic comparison of inbreeding depression between maize and its wild relative teosinte. The major results are:
1. Inbreeding depression is generally greater in maize than teosinte considering the same traits.
2. Inbreeding increases the genetic variance, but not as much as you would expect from simple genetic models.
3. The authors develop and apply a new method to identify QTL representing large-effect rare variants carried by only a single parent. They find more of these loci in teosinte than maize, which is consistent with origin hypotheses for maize.
4. The differing variance component estimates between maize and teosinte suggest that the former species has a "mostly polygenic, small-effect recessive variation" causing inbreeding depression while the latter species has a greater contribution from larger-effect variants.

There is a lot of interesting data and analyses in this paper. Figure 3 is particularly striking – there is a very strong positive relationship between the mean phenotype of outcrossed and selfed progeny produced by the same genotype. This level of correlation has not been observed in breeding designs from other species involving both selfed and outcrossed progeny. Second, the "Rare allele scan" seems to be an important methodological development from this paper. Previous work in a few systems has parsed the total mutation load into lethal/sterile mutations and everything else. The RAS provides a useful partition of the "everything else" collection into major but sublethal loci and everything else. I think this method is original to this paper. If that is correct, RAS should be punched up a bit more in a revised draft as a broadly useful technique for future studies. For example, Figure S1 (Diagram of key features of RAS) would probably be a better thing to include in the main paper than the current Fig 1 (which is very hard to understand).

Three major revisions would greatly improve the paper: (a) text changes for readability, (b) clarification of the genetic model and how parameters are estimated, and (c) a more explicit argument about how inbreeding variance component estimates justify the arguments about the loci that explain inbreeding depression (result 4 listed above).

(a) After reading only the Introduction, I could not follow the Results and Discussion which immediately follow the Intro. I had to skip to the Methods and read that, then read the Yang et al paper from PNAS in 2019, then Chen et al (2020), and then read the Supplemental materials from all three papers. After that, I could mostly follow the Results. I think the gentle reader is going to need much better preparation in a revised Introduction. Yang et al (2019) and Chen et al (2020) analyze the same experiment as this study. A review of these papers would be a good long paragraph for the introduction. What did the prior papers show? What did they not show that is addressed here?

(b) The Yang et al (2019) paper estimated genetic variance components, but I think they must have used a different model than applied here. This needs to be made clear. The current paper is applying the C&W parameterization for single locus effects (Cockerham, 1983; Cockerham & Weir, 1984; Weir & Cockerham, 1977) with the assumptions of bi-allelic loci, no epistasis, no

LD among QTLs, and that all parents in the breeding design were fully outbred. In this case, the covariances among relatives are functions of 4 parameters: Va, Vd, D1 and D2 (e.g. pg 19 of ms). Yang et al (2019) include the inbred progeny in the analysis but do not have D1 and D2 as outputs. Perhaps D1 and D2 were assumed to equal zero? If so, the present analysis is a definite upgrade because the only way to have Vd > 0 while D1 = D2 = 0 is for the two alleles to be equally frequent at all QTL. This is not a realistic assumption.

A second thing to clarify is exactly what is being estimated in the current ms. The authors fit an agricultural style glm (bottom of pg 16) and extract 'observational variance components' like specific and general combining ability, then estimate 'causal components', e.g. Va and Vd, by formulas relating components (bottom of pg 18). However, it is not clear that D1 and D2 are separately estimated. In principle, they should be identifiable because the variance among selfed families depends on both D1 and D2 while the covariance of outcrossed progeny with their selfed sibs depends only on D1 and Va. However, perhaps this is impractical here? If the authors intend to use (D1+D2/8) as an aggregate statistic for genetic inference, that should be stated explicitly. If they could be distinguished, D1 in teosinte could be compared to D1 in maize. The rare recessive alleles found from the RAS scan should be big positive contributors to D1 in teosinte producing a difference (hints to this effect at bottom of pg 10).

A third thing to clarify: The prior papers use GBS genotyping (and resulting relatedness matrix) for variance component estimation while the present paper apparently does not. Here, variance comps come from the overall resemblance of relatives of different types. The estimates for Va seem to agree OK (correlations on pg 9), but we need a reason why the relatedness matrix is not employed here. Is there a conceptual difficulty or is it just than nobody has written code to do marker-based identity-by-state calculations to address the additional identity-by-descent relationships that emerge with inbreeding (Cockerham, 1971)? The authors are using 'realized variation' inbreeding values estimated from markers on pg 8.

(c) The authors nicely explain how D1 depends on dominance and allele frequency on pg 11 (also Figure S6). The basic difficulty is that you need the (partially) recessive allele to be more frequent than the alternative to get D1 < 0, which is not a popular model for inbreeding depression. As noted, pseudo-overdominance on intermediate frequency polymorphisms can resolve this issue by generating D1 << 0. While this text is clear, but I am not sure how it leads to the summary in the Abstract: "These results suggest a mixture of mostly polygenic, small-effect recessive variation underlying inbreeding depression … in maize"
What is "recessive variation" in relation to the arguments on pg 11?
Recessive variation sounds like the classical dominance model with inbreeding depression caused by rare (partially) recessive alleles. Rare recessives predict D1 > 0, and if these loci are the sole contribution to variation, D1 > Va. Along these lines, I think it is worth noting that much of the genetic variance in some of these traits may not be contributing to inbreeding depression.

Cited:
Cockerham C. 1971. Higher order probability functions of identity of alleles by descent. *Genetics* **69:** 235-246.

Cockerham CC. 1983. Covariances of relatives from self-fertilization. *Crop science* **23:** 1177-1180.
Cockerham CC, Weir BS. 1984. Covariances of relatives stemming from a population undergoing mixed self and random mating. *Biometrics* **40:** 157-164.
Weir BS, Cockerham CC. 1977. Two-locus theory in quantitative genetics. In: Pollack E, Kempthorne O and Bailey TB, eds. *Proceedings of the international conference on quantitative genetics*. Ames, Iowa.: Iowa State University Press. 247-262.

John Kelly
Lawrence, KS
September 24, 2021
