# Supplementary material for: Domestication reshaped the genetic basis of inbreeding depression in a maize landrace compared to its wild relative, teosinte
Source: PLoS Genet. 2021 Dec 20;17(12):e1009797. doi: 10.1371/journal.pgen.1009797 (PMC8722731; doi:10.1371/journal.pgen.1009797)
Supplement: Attachment — Submitted filename: Review PLoS-Genetics-MaizeTeosinteID.docx [file pgen.1009797.s042.docx]

Review of:	PGENETICS-D-21-01141	Samayoa et al.

“Domestication Reshaped the Genetic Basis of Inbreeding Depression in a Maize Landrace Compared to its Wild Relative, Teosinte“

The paper compares levels of inbreeding depression (ID) for 18 traits and details of their genetic architecture between a local landrace of maize in Mexico and its wild relative teosinte. Given that this domesticated maize landrace has experienced one or more population bottlenecks and some inbreeding, we expect the inbreeding to have exposed the mostly recessive deleterious alleles that make up most of the segregating load to purifying selection. However, instead of being purged of much of its segregating load, the maize landrace displays substantially higher ID (segregating load) for many traits than the ancestral (and more outcrossing) teosinte population. This is particularly true for the reproductive traits most directly related to fitness where maize has 2-3x as much ID as teosinte. This increase in the load is surprising, given that purging appears to have occurred based on the results presented that the maize landrace appears to have only about half the frequency of segregating lethals and sub-lethals relative to this teosinte population (with each thus having bigger effects). Likewise, the number of regions with significant segregation distortion is far lower in the maize – yet the three regions of SD found in three selfing families in maize (Fig. 6) are all in highly restricted genomic locations associated with high ID (Fig. S22).

Another surprising result was the lower than expected genetic variation among self-fertilized families based on measured levels of additive and dominance variance implying that additive and homozygous dominance effects limits the variation available to selection under partial inbreeding. The authors conclude that the rare large-effect variants important in teosinte were depleted in maize, leaving a residue of polygenic “harder to purge” recessive alleles with individually small, but cumulatively large, effects. They leave unresolved, however, why the “segregating mutational burden” (ID) increased in maize compared to teosinte – which is not emphasized or discussed much.

The paper’s important results include not only the finding of higher ID in maize despite having fewer strongly deleterious alleles but also that the genetic variance is primarily additive in both populations; the fact that inbred maize progeny lose homeostasis; the absence of synergistic epistasis for ID in maize (despite some in teosinte); and that ID declines in families of higher parental breeding value (fitness), as inferred from the outcrossed progeny.

Overall, I was impressed with this paper. The approach and questions are clear, the experimental design and sample sizes are excellent, a variety of powerful methods are brought to bear (all fully described) on a large amount of high-quality data, and the results are presented clearly and convincingly. The figures were carefully prepared and are richly informative. The authors ably summarize an immense amount of data, filling a large number of supplementary tables and figures. The authors also place their results squarely in appropriate context citing the relevant literature. I also liked their careful consideration of alternative hypotheses, e.g. that loci affecting seed germination could affect their SDR results (p. 14, Fig. S24). Thus, my suggestions are few and aimed at improving an already strong paper.

As already mentioned, the authors do a good job of documenting and explaining the purging of strongly deleterious mutations in maize relative to teosinte. They leave a gap, however, in not accounting for why maize carries more weakly deleterious mutations than its relative. This coincides with the average frequency of derived alleles being higher in maize relative to teosinte (Figure 2D). While drift of weakly selected alleles in small or inbred populations might account for some of this, the strong overdominant selection documented in Fig. S22 associated with regions of low recombination suggests that the many individually weak alleles may be aggregated into localized genomic blocks, generating strong pseudo-overdominance (POD). The authors acknowledge that the “pseudo- overdominance generated by repulsion phase linkages among deleterious variants appears to be widespread (Graham et al. 1997; Garcia et al. 2008; Larièpe et al. 2012; Yang et al. 2017a), and could produce the negative covariances between additive and homozygous dominance deviations observed in this study.” Can the authors suggest any way to test this conjecture?

Given these points, the authors may wish to revisit their statement on p. 15: “Our inbreeding depression QTL are detected with limited genomic resolution, such that the QTL intervals used to define regions in which to measure rare allele load probably contain too many neutral variants for us to detect any relationships.” They may also wish to consider the alternative hypothesis that much of the ID, particularly in the bottlenecked / inbred maize population, may derive from specific local POD regions rather than from simple segregation (sustaining deleterious alleles at higher frequencies, as they observe). Such regions could impose strong selection, sustaining the load even in the face of inbreeding and drift. It would also shelter newly occurring mutations within such regions from selection, helping to account for how load could have increased in maize. Additional support for POD comes from their site frequency spectra finding that maize has more intermediate frequency derived alleles relative to teosinte (Fig. 2).

Minor comment:

Fig. S22 – I only count 4 SDRs with a deficiency of heterozygotes – not the 5 claimed in the first full paragraph on p. 14.
