# Supplementary material for: Domestication reshaped the genetic basis of inbreeding depression in a maize landrace compared to its wild relative, teosinte
Source: PLoS Genet. 2021 Dec 20;17(12):e1009797. doi: 10.1371/journal.pgen.1009797 (PMC8722731; doi:10.1371/journal.pgen.1009797)
Supplement: Attachment — Submitted filename: Inbreeding depression response to reviewers.docx [file pgen.1009797.s043.docx]

**Editor Comments:**

This is an important and interesting paper, suitable for a wide audience, and hence appropriate for PLoS Genetics. None of the three reviewers had any serious technical concerns (but see below for some suggestions), but they did express concerns in terms of the readability of the current ms for a general audience. Hence the recommendation of a major revision.

Response: Thanks for these generous comments, we have made major changes to the presentation in the revised version, cutting some details from the results section and beefing up the introduction to better frame the paper as detailed below.

A couple of suggestions beyond those offered by the reviewers. First, the quadratic components under inbreeding are a dark art for even most quantitative geneticists, and the notation varies widely even among its practitioners (W&L Table 11.2). That can make discussions hard to follow. Might I suggest that instead of D1 and D2, you use sigma_{ADI} and sigma_{DI} which have a bit more widespread (and also suggest what is being measured), and then define these (W&L Table 1), e.g., D1 = sigma(alpha_ki, delta_kii), the association between the additive effect of allele i (at locus k) and its dominance deviation (ii). While you do clearly say this, its a bit hard to follow unless you already know this, whereas I find the equation definition is usually clearer (and less ambiguous).

We changed the nomenclature as suggested.

Reviewer 3 notes that there are a lot of things going on in the ms, and to a reader outside the field, their interconnections may not be entirely clear, making the discussion a bit hard to follow (although the authors do nicely connect the dots at the end). In parts, it does feels a bit like a six pack of beer without the plastic rings that holds all the cans together. I think this can be a bit more clearly addressed with a short paragraph up front of what the paper will be looking for, and how these connect with each other (e.., variance components, rare QTLs, etc.).

We dropped most of the discussion of changes in variance under inbreeding (because our estimates of this phenomenon were not precise), which helped to simplify the paper. We’ve rewritten the introduction to better frame the paper, along the lines of the suggestions of reviewer 1.

Another presentation suggestion is for the rare haplotype analysis (essentially a single allele test) to display the model in a slightly different from, y_ij = + x_{haij}*alpha + e_{ij}, where (new line) = all of the other stuff. The critical term can get buried in all of the model effects, whereas if they are clearly separated, the model becomes a bit more straightforward to an average reader.

We’ve done this, separating the equation into two lines, with the first line showing the haplotype additive and dominance and effects and the second line having everything else.

Possible other things to consider
Some connection with heterosis would be interesting. It can be the flip side of inbreeding depression, or something entirely different. In particular I think a little more discussion on the role of loci around centromeres generating pseudo-overdominace might also be in order (a comment also suggested by reviewer #2)

We added the following to the first paragraph of the introduction to make the connection (and some distinction) between inbreeding depression and heterosis clear:

“Inbreeding depression is closely related to the inverse phenomenon of hybrid vigor (heterosis), which is exploited to make high-yielding modern maize varieties (Holland 2009). Inbreeding depression typically refers to reduced vigor resulting from inbreeding within local populations whereas heterosis refers to increased vigor in crosses between differentiated populations. Thus, inbreeding depression evolves differently than heterosis in response to drift (Oakley *et al.* 2019) and epistasis has distinct effects on the two phenomena (Lynch and Walsh 1998). Nevertheless, much of the genetic basis for both inbreeding depression and heterosis involves at least partially recessive deleterious variants whose effects are expressed when homozygous in inbreds or masked by dominant favorable alleles in hybrids (Charlesworth and Charlesworth 1999; Charlesworth and Willis 2009; Yang *et al.* 2017a). Overdominance at a subset of loci contributing a small amount of variation cannot be ruled out, but repulsion-phase linkage and low recombination between recessive deleterious mutations can generate pseudo-overdominance or associative overdominance at the level of haplotypes, contributing to the retention of deleterious mutations (Becher *et al.* 2020; Gilbert *et al.* 2020).”

We added the following to the result and discussion section to point out the important role of reduced recombination and pseudo-overdominance:

“Pseudo-overdominance generated by repulsion phase linkages among deleterious variants appears to be widespread in maize (Graham *et al.* 1997; Garcia *et al.* 2008; Larièpe *et al.* 2012; Yang *et al.* 2017a) and in particular, genomic regions with low recombination rates can shelter deleterious variants dispersed in repulsion phase (McMullen *et al.* 2009), permitting a buildup of genetic load that may have initially accumulated through drift (Waller 2021). ”

**Comments to the Authors:**

**Reviewer #1**:

“Domestication Reshaped the Genetic Basis of Inbreeding Depression in a Maize Landrace Compared to its Wild Relative, Teosinte” by Holland et al.

Holland et al present a detailed quantitative genetic comparison of inbreeding depression between maize and its wild relative teosinte. The major results are:

1. Inbreeding depression is generally greater in maize than teosinte considering the same traits.

2. Inbreeding increases the genetic variance, but not as much as you would expect from simple genetic models.

3. The authors develop and apply a new method to identify QTL representing large-effect rare variants carried by only a single parent. They find more of these loci in teosinte than maize, which is consistent with origin hypotheses for maize.

4. The differing variance component estimates between maize and teosinte suggest that the former species has a “mostly polygenic, small-effect recessive variation” causing inbreeding depression while the latter species has a greater contribution from larger-effect variants.

There is a lot of interesting data and analyses in this paper. Figure 3 is particularly striking – there is a very strong positive relationship between the mean phenotype of outcrossed and selfed progeny produced by the same genotype. This level of correlation has not been observed in breeding designs from other species involving both selfed and outcrossed progeny. Second, the “Rare allele scan” seems to be an important methodological development from this paper. Previous work in a few systems has parsed the total mutation load into lethal/sterile mutations and everything else. The RAS provides a useful partition of the “everything else” collection into major but sublethal loci and everything else. I think this method is original to this paper. If that is correct, RAS should be punched up a bit more in a revised draft as a broadly useful technique for future studies. For example, Figure S1 (Diagram of key features of RAS) would probably be a better thing to include in the main paper than the current Fig 1 (which is very hard to understand).

Thanks for these suggestions. We moved the rare allele scan description figure to Figure 1. We relegated the previous Figure 1 to supplemental S1 and made a new, much simpler figure demonstrating the distribution of outcross and selfed progeny values for one trait of each category from maize and teosinte, which illustrates the effect of inbreeding on the trait distributions in each population (new Figure 2).

Three major revisions would greatly improve the paper: (a) text changes for readability, (b) clarification of the genetic model and how parameters are estimated, and (c) a more explicit argument about how inbreeding variance component estimates justify the arguments about the loci that explain inbreeding depression (result 4 listed above).

(a) After reading only the Introduction, I could not follow the Results and Discussion which immediately follow the Intro. I had to skip to the Methods and read that, then read the Yang et al paper from PNAS in 2019, then Chen et al (2020), and then read the Supplemental materials from all three papers. After that, I could mostly follow the Results. I think the gentle reader is going to need much better preparation in a revised Introduction. Yang et al (2019) and Chen et al (2020) analyze the same experiment as this study. A review of these papers would be a good long paragraph for the introduction. What did the prior papers show? What did they not show that is addressed here?

(1) We rewrote the paper extensively to make it easier to follow. We appreciate the comment that we did not really frame the paper well in light of our previous work on these same populations, so we’ve added quite a bit on that in the introduction and it helps clarify what we are doing here that is new. Thanks for this advice.
(2) Part of the rewriting included explicitly contrasting the new quantitative genetics analysis compared to the previous Yang et al paper up front in the introduction (Lines 112-125). That should make this clearer.
(3) We mostly removed any emphasis on the covariance components under inbreeding, so some of the discrepancies noted are not an issue (more on this below; the poor precision of the estimates is now noted).

(b) The Yang et al (2019) paper estimated genetic variance components, but I think they must have used a different model than applied here. This needs to be made clear. The current paper is applying the C&W parameterization for single locus effects (Cockerham, 1983; Cockerham & Weir, 1984; Weir & Cockerham, 1977) with the assumptions of bi-allelic loci, no epistasis, no LD among QTLs, and that all parents in the breeding design were fully outbred. In this case, the covariances among relatives are functions of 4 parameters: Va, Vd, D1 and D2 (e.g. pg 19 of ms). Yang et al (2019) include the inbred progeny in the analysis but do not have D1 and D2 as outputs. Perhaps D1 and D2 were assumed to equal zero? If so, the present analysis is a definite upgrade because the only way to have Vd > 0 while D1 = D2 = 0 is for the two alleles to be equally frequent at all QTL. This is not a realistic assumption.

Correct, the Yang et al (2019) paper included additive and dominance genetic relationships and estimated variance components associated with those effects, ignoring the additional components of variance that are introduced under inbreeding. We added this information to the introduction and this helped to make it clear that what we did with the diallel model allowed us to relax that (and some other) assumptions (Lines 160-175).

A second thing to clarify is exactly what is being estimated in the current ms. The authors fit an agricultural style glm (bottom of pg 16) and extract ‘observational variance components’ like specific and general combining ability, then estimate ‘causal components’, e.g. Va and Vd, by formulas relating components (bottom of pg 18). However, it is not clear that D1 and D2 are separately estimated. In principle, they should be identifiable because the variance among selfed families depends on both D1 and D2 while the covariance of outcrossed progeny with their selfed sibs depends only on D1 and Va. However, perhaps this is impractical here? If the authors intend to use (D1+D2/8) as an aggregate statistic for genetic inference, that should be stated explicitly. If they could be distinguished, D1 in teosinte could be compared to D1 in maize. The rare recessive alleles found from the RAS scan should be big positive contributors to D1 in teosinte producing a difference (hints to this effect at bottom of pg 10).

We did estimate D1 and D2 (Cov(ADI) and V(DI) in the new terminology) from the diallel model, however the precision of these estimates was not good: Cov(ADI) was significant (more than 2 SE away from zero) for only two traits in each population, and similar for V(DI). Therefore, in the previous version we did not mention this and instead chose to focus on the ratio of observed to expected variance among selfed families, as that is estimated with a bit more precision (a total of 9 pop-trait combinations are significantly different from 1), and we thought this ratio leads to a more obvious interpretation regarding the expected effectiveness of selection under inbreeding. However, considering these comments and the comments from reviewer #3 (which pushed us to demonstrate statistical significance), we find that although there is a non-random trend toward less than expected variance in the selfed progenies, the reduction is rarely significant for individual traits. We added a new Table S6 showing the estimates of these covariance components, their standard errors, and indicating whether they are significant at p = 0.05. Considering the relatively poor precision of our estimates of Cov(ADI) and V(DI) and even the ratio of observed to expected genetic variance among selfed families, we decided to remove most of this from the paper. Instead, we now mention in the methods section explicitly how we estimated Cov(ADI) and V(DI), then in the results just say that these were estimated with poor precision, and now we leave it at that. We now feel it may be risky to make too much biological inference from these imprecise parameter estimates.

A third thing to clarify: The prior papers use GBS genotyping (and resulting relatedness matrix) for variance component estimation while the present paper apparently does not. Here, variance comps come from the overall resemblance of relatives of different types. The estimates for Va seem to agree OK (correlations on pg 9), but we need a reason why the relatedness matrix is not employed here. Is there a conceptual difficulty or is it just than nobody has written code to do marker-based identity-by-state calculations to address the additional identity-by-descent relationships that emerge with inbreeding (Cockerham, 1971)? The authors are using ‘realized variation’ inbreeding values estimated from markers on pg 8.

You are correct that it in theory it is possible to estimate all of the different single-locus identity-by-descent coefficients using sequence data. Ackerman et al (Genetics; 2017) developed this method and have released software mpgd to do this. We investigated this software but decided not to use it here because it is computationally very intensive (even with a cluster, it seems like it would be running for months, perhaps a year or more, to estimate all the coefficients for all ~8000 individuals from our experiment), its robustness to the high levels of missing data associated with the very low GBS coverage in our data has not been reported (but we expect that could be a serious problem), and even if we are able to get all of the different relationship coefficients estimated, we expect to hit problems with mixed model convergence when we fit them all together. I’m quite interested in following up on this and expect we could work around these issues, but am certain it will take a long time (years) to sort out. We addressed this possibility in the revised manuscript as follows (Line 318):

A method to use population sequence data to estimate higher-order identity-by-descent coefficients has been developed (Ackerman *et al.* 2017) and could be used to more directly estimate higher-order covariance components, but this approach is very computationally intensive and will be investigated in future efforts.

(c) The authors nicely explain how D1 depends on dominance and allele frequency on pg 11 (also Figure S6). The basic difficulty is that you need the (partially) recessive allele to be more frequent than the alternative to get D1 < 0, which is not a popular model for inbreeding depression. As noted, pseudo-overdominance on intermediate frequency polymorphisms can resolve this issue by generating D1 << 0. While this text is clear, but I am not sure how it leads to the summary in the Abstract: “These results suggest a mixture of mostly polygenic, small-effect recessive variation underlying inbreeding depression … in maize”

We tried to clarify this statement (Line 37) as:

“These results suggest a mixture of mostly polygenic, small-effect partially recessive effects in linkage disequilibrium underlying inbreeding depression…”

We are implying that the individual variant gene action effects involve partial dominance/recessive gene action, which can be reconciled with apparent overdominance via linkage and pseudo-overdominance. And, further, that the individual QTL effects are relatively small contributors to the overall variance; most of the action appears to be polygenic partial dominance/recessive. In the revised version, we are also backing off from making inferences about and from the D1 (Cov(ADI) parameter, so the need to invoke strong pseudo-overdominance is lessened.

What is “recessive variation” in relation to the arguments on pg 11?

We use both “recessive variation” and “dominance variation” in the paper, depending on whether the inference is toward an unfavorable allele (which tend to be partially recessive) or a more favorable allele (which tend to be partly dominant).

Recessive variation sounds like the classical dominance model with inbreeding depression caused by rare (partially) recessive alleles. Rare recessives predict D1 > 0, and if these loci are the sole contribution to variation, D1 > Va. Along these lines, I think it is worth noting that much of the genetic variance in some of these traits may not be contributing to inbreeding depression.

Right, this is what we are implying. And you are correct that we expect D1 > 0 if most recessive unfavorable alleles are rare. Which is why we were troubled by the negative D1 estimates and hypothesized that pseudo-overdominance was generated by linked rare recessives. Again, backing off from making inferences about D1 at least relieves us of this problem (although I should note that all the other maize studies, in US breeding populations, have resulted in negative estimates of D1…so it’s really a thing, I just don’t fully understand it).

Regarding the last point, we have added this sentence to the abstract that reflects the idea that much of the genetic variance does not contribute to inbreeding depression: “Parental breeding values were highly consistent between outcross and selfed offspring, indicating that additive effects determine most of the genetic value even in the presence of strong inbreeding depression”

Cited:

**Cockerham C. 1971.** Higher order probability functions of identity of alleles by descent. *Genetics* **69:** 235-246. **Cockerham CC. 1983.** Covariances of relatives from self-fertilization. *Crop science* **23:** 1177-1180.

**Cockerham CC, Weir BS. 1984.** Covariances of relatives stemming from a population undergoing mixed self and random mating. *Biometrics* **40:** 157-164.

**Weir BS, Cockerham CC. 1977.** Two-locus theory in quantitative genetics. In: Pollack E, Kempthorne O and Bailey TB, eds. *Proceedings of the international conference on quantitative genetics*. Ames, Iowa.: Iowa State University Press. 247-262.

John Kelly

Lawrence, KS

**Reviewer #2:**

The paper compares levels of inbreeding depression (ID) for 18 traits and details of their genetic architecture between a local landrace of maize in Mexico and its wild relative teosinte. Given that this domesticated maize landrace has experienced one or more population bottlenecks and some inbreeding, we expect the inbreeding to have exposed the mostly recessive deleterious alleles that make up most of the segregating load to purifying selection. However, instead of being purged of much of its segregating load, the maize landrace displays substantially higher ID (segregating load) for many traits than the ancestral (and more outcrossing) teosinte population. This is particularly true for the reproductive traits most directly related to fitness where maize has 2-3x as much ID as teosinte. This increase in the load is surprising, given that purging appears to have occurred based on the results presented that the maize landrace appears to have only about half the frequency of segregating lethals and sub-lethals relative to this teosinte population (with each thus having bigger effects). Likewise, the number of regions with significant segregation distortion is far lower in the maize – yet the three regions of SD found in three selfing families in maize (Fig. 6) are all in highly restricted genomic locations associated with high ID (Fig. S22).

Another surprising result was the lower than expected genetic variation among self-fertilized families based on measured levels of additive and dominance variance implying that additive and homozygous dominance effects limits the variation available to selection under partial inbreeding. The authors conclude that the rare large-effect variants important in teosinte were depleted in maize, leaving a residue of polygenic “harder to purge” recessive alleles with individually small, but cumulatively large, effects. They leave unresolved, however, why the “segregating mutational burden” (ID) increased in maize compared to teosinte – which is not emphasized or discussed much.

We added the following paragraph to the results section regarding WHY the segregating burden has become greater in maize (Lines 232-247):

“Why is the mean segregating genetic burden greater in this maize population than its closest wild relative population? Our results suggest that although maize has fewer deleterious variants than teosinte (due to an overall reduction in diversity following the domestication bottleneck), they occur at higher frequency in maize than in teosinte, increasing the mean segregating burden. Simons et al. (2014) found that population bottlenecks followed by growth are expected to decrease the fixed burden and increase the segregating burden due to weak recessive deleterious alleles, suggesting that our observed results can largely be explained by drift resulting from the domestication bottleneck. In addition, pseudo-overdominance generated by repulsion phase linkages among deleterious variants appears to be widespread in maize (Graham *et al.* 1997; Garcia *et al.* 2008; Larièpe *et al.* 2012; Yang *et al.* 2017a) and in particular, genomic regions with low recombination rates can shelter deleterious variants dispersed in repulsion phase (McMullen *et al.* 2009), permitting a buildup of genetic load that may have initially accumulated through drift (Waller 2021). In addition, Chen et al. (2020) suggested that selection coefficients for many traits in maize could be lower than in teosinte because human selection is often aimed at traits other than the domestication-related traits measured in this study, and because the structure of trait genetic correlations in maize results in generally lower correlations of most traits with fitness. ”

The paper’s important results include not only the finding of higher ID in maize despite having fewer strongly deleterious alleles but also that the genetic variance is primarily additive in both populations; the fact that inbred maize progeny lose homeostasis; the absence of synergistic epistasis for ID in maize (despite some in teosinte); and that ID declines in families of higher parental breeding value (fitness), as inferred from the outcrossed progeny.

Overall, I was impressed with this paper. The approach and questions are clear, the experimental design and sample sizes are excellent, a variety of powerful methods are brought to bear (all fully described) on a large amount of high-quality data, and the results are presented clearly and convincingly. The figures were carefully prepared and are richly informative. The authors ably summarize an immense amount of data, filling a large number of supplementary tables and figures. The authors also place their results squarely in appropriate context citing the relevant literature. I also liked their careful consideration of alternative hypotheses, e.g. that loci affecting seed germination could affect their SDR results (p. 14, Fig. S24). Thus, my suggestions are few and aimed at improving an already strong paper.

As already mentioned, the authors do a good job of documenting and explaining the purging of strongly deleterious mutations in maize relative to teosinte. They leave a gap, however, in not accounting for why maize carries more weakly deleterious mutations than its relative. This coincides with the average frequency of derived alleles being higher in maize relative to teosinte (Figure 2D). While drift of weakly selected alleles in small or inbred populations might account for some of this, the strong overdominant selection documented in Fig. S22 associated with regions of low recombination suggests that the many individually weak alleles may be aggregated into localized genomic blocks, generating strong pseudo-overdominance (POD). The authors acknowledge that the “pseudo- overdominance generated by repulsion phase linkages among deleterious variants appears to be widespread (Graham et al. 1997; Garcia et al. 2008; Larièpe et al. 2012; Yang et al. 2017a), and could produce the negative covariances between additive and homozygous dominance deviations observed in this study.” Can the authors suggest any way to test this conjecture?

See our response to the previous comment from reviewer #2 on accounting for why maize carries more segregating burden than teosinte. Since we have removed any focus on the negative estimates of covariances between additive and dominance effects in inbred individuals, we have not included any additional discussion of how to investigate those effects. We believe the question of whether there is widespread pseudo-overdominance for yield-related traits in maize is mostly settled, the evidence for this is abundant (such as in the papers cited).

Given these points, the authors may wish to revisit their statement on p. 15: “Our inbreeding depression QTL are detected with limited genomic resolution, such that the QTL intervals used to define regions in which to measure rare allele load probably contain too many neutral variants for us to detect any relationships.” They may also wish to consider the alternative hypothesis that much of the ID, particularly in the bottlenecked / inbred maize population, may derive from specific local POD regions rather than from simple segregation (sustaining deleterious alleles at higher frequencies, as they observe). Such regions could impose strong selection, sustaining the load even in the face of inbreeding and drift. It would also shelter newly occurring mutations within such regions from selection, helping to account for how load could have increased in maize. Additional support for POD comes from their site frequency spectra finding that maize has more intermediate frequency derived alleles relative to teosinte (Fig. 2).

Thanks for these comments, we have modified the statement that was on p. 15 to read:

“Our inbreeding depression QTL are detected with limited genomic resolution, which hinders detection of relationships between predicted load within localized genomic regions and phenotypic inbreeding depression.” (Line 487)

In other words, we are not interpreting the lack of relationship between localized load and QTL effects as strong evidence that there is no underlying relationship, rather, we are suggesting that our data don’t lead to a good way to test this relationship.

We have added some of the ideas about POD in this comment to the section discussing the higher segregating load in maize, as already mentioned above in the paragraph starting: “Why is the mean segregating genetic burden greater in this maize population than its closest wild relative population? … ”

Minor comment:

Fig. S22 – I only count 4 SDRs with a deficiency of heterozygotes – not the 5 claimed in the first full paragraph on p. 14.

We double-checked this, there are five such cases that can be seen in the figure (although two of them are partly overlapping so perhaps why they are difficulty to see).

**Reviewer #3:**

The authors investigate genetic load and inbreeding depression in a pair of maize-teosinte populations evaluated for 18 traits using quantitative genetic theory and rare allele scans.
The research topic is very important and interesting for a broad readership, the data are unique, the analyses are statistically sound and innovative, the results are interesting.
The paper is very hard to follow and some paragraphs require deep knowledge of the subject to follow the line of argument.

We appreciate the criticism that the paper was hard to follow and have worked hard to improve that aspect substantially.

The paper presents many correlations in the text as on page 9: “Narrow-sense heritability estimates based on additive genetic variances were highly correlated (r = 0.85 in teosinte and 0.83 maize) between the previous analysis of realized relationships and the current diallel model.
In many cases it is not clear where to find these results, what is expected, what is their relevance (two times same result?) and in general correlations do not say much as a high correlation can originate from all sorts of data structures.

A lot of this sort of text was to demonstrate that the parameter estimates from the diallel model were generally in line with previous estimates made a different way, to support the results of this new analysis. But we agree, it’s too much text, so we’ve cut this specific sentence and a number of similar comparisons between different ways of estimating the same parameters. We also dropped the old Tables S5 and S6 which had all of this correlation information.

Some results are confounded for example on page 9: “The cases of decreased
environmental variance may occur due to reduced scale associated with inbreeding, whereas the
increased environmental variance observed for maize vegetative and reproductive traits suggests that partially inbred maize plants have reduced capacity for homeostasis.”
How can these two factors be separated? The authors are careful with their conclusions but they still seem rather speculative.

We cannot separate these factors, but increased residual variance associated with inbreeding in maize (and a decrease in trait mean) cannot be due to scale, since they are in opposite directions. We rewrote this paragraph to make our meaning clearer:

“Separate estimation of the residual non-genetic variation associated with selfed vs. outcrossed progenies permits a test of the hypothesis that outbred and inbred plants differ for environmental variance (Falconer and Mackay 1996; Whitlock and Fowler 1999). Our results demonstrate that environmental variance increased with inbreeding on average for maize vegetative and reproductive traits (mean increases of 40% and 20%, respectively), suggesting that partially inbred maize plants have reduced capacity for homeostasis for these traits. In contrast, residual environmental variance decreased on average with inbreeding for maize environmental response traits (mean decrease of 39%) and all groups of teosinte traits (decreased from 3% to 29%); these reductions may occur due to reduced scale associated with inbreeding.”

The results are not sufficiently discussed and interpreted for example the results in Figure 3 and the paragraph on page 10 “The very high correlations between outbred and inbred progeny…at the same time.” Results are very important and have direct implications for the utilization of landraces or other genetic resources for breeding. The authors do not put this into context but leave it to the reader to make something of it.

We expanded on this point in the revision: “Thus, it appears that some important components of the genetic architecture of inbreeding depression in maize differ between landrace and modern breeding populations, due to their different histories of population management and selection.” (Line 329) Also, by cutting an intervening paragraph, we connect this part to the previous result paragraph to make the connections/comparisons between landrace and breeding populations clearer. In general, we tried to beef up the interpretation and reduce the detailed minor results in the text.

The prediction of genetic variance among selfed families requires a more profound discussion. Variance components are generally not very precise, especially dominance variances. I suspect that “less than 90% expected variance” is not significantly different from 100%. In addition, averaged across traits it looks pretty close to 100%. A trait like tillering is probably not normally distributed and this can easily result in deviations from what is expected.

The reviewer is correct that the variance components estimates are not very precise. To address the significance of the higher-order variance components, we constructed approximate Z-tests from the ratio of the estimators to their standard errors, considering ratios greater than 2 to indicate significant differences from zero. Only a few estimates of C(ADI) and V(DI) were significant this way, but we were surprised that no dominance variance components for any trait in either population were significant by this method. This test has low power, so it’s hard to make much inference from this. Unfortunately, we have been unable to successfully construct likelihood ratio tests for the C(ADI) and V(DI) components. To test C(ADI), for example, we attempted to fit a reduced model that constrains C(ADI) to 2*GCA variance, which is the expectation when C(ADI) is zero, but this constrained model led to singularities in the AI matrix and we have been unable to get convergence. So, we are a bit stuck at this point.

Therefore, we removed the text relating to the less than expected genetic variance among selfed families and replaced it with a couple of sentences indicated that we estimated these other components of variance, but they had poor precision and left it at that in the manuscript.

The scan for rare allele effects suffers from the same limitations as QTL mapping and GWAS. QTL are not reproducible across populations for many reasons (e.g. power etc.), effect estimates are biased especially for rare alleles. Effects need to be validated in an independent sample. The authors ignore these limitations and it is questionable if their conclusions are justified. Shouldn't effects in teosinte be predictive for maize if they share genomic regions? Wouldn't that be a nice validation?

Our method uses IBD within a population to ‘join’ alleles in the analysis. Thus, we don’t yet have a way to join alleles from different populations (as here we define IBD with respect to the two different reference populations). However, we agree that some sort of validation would be a useful indicator of the reliability of these models. Therefore, we performed 10-fold cross-validation of the rare allele scan models, training effect estimates in 90% of the data set and using those estimates to predict phenotypes of the held-out 10% of individuals in the test set each time. We have included these new results in the paper (Table S13), as they indicate that the rare allele scan models almost always increase the prediction ability in validation sets over the polygenic background-only model (using the principal components without QTL). Although the boosts in prediction ability were generally not very big, our expectation is that these QTL are necessarily limited in their ability to explain total population variance because each QTL is by definition restricted to the effects of a single parental haplotype (and thus occurring about 1% on average in the population). And as already mentioned in the paper, most of the variation appears to be polygenic in nature (and that is captured in our baseline model…which also can absorb part of the effects of these QTL on family differences). We believe the real power of these QTL is to better understand the otherwise unexpected effects observed in specific selfed progeny families beyond what the polygenic background model suggests.

My impression is that the paper would have profited a lot from a clear focus. At the moment it is more like an assembly of results that are not well connected by a common hypothesis or research question. The manuscript is also lacking interpretation of results beyond the analysis of inbreeding depression in a specific pair of populations. Grouping of the traits helped, but it would have been better to focus on a few traits. The data have been presented in other publications so no need to use all traits. The paper becomes rather unwieldy with many supporting figures and tables.

We have worked hard to tie the paper together better and reducing focus on some previous issues like the S1 family variance has helped. We feel that some of the power of the study is that we have measured many traits, giving us a good sampling of genetic architectures that we are trying to summarize over. To help focus the basic results, we dropped the old Figure 1 that included inbreeding depression estimates for all 18 traits and created a new Figure 2 that illustrates the inbreeding depression effects using only three traits.
